# Prevalence and care index of early childhood caries in mainland China: evidence from epidemiological surveys during 1987–2013

**DOI:** 10.1038/srep18897

**Published:** 2016-01-13

**Authors:** Xiaonan Zhang, Sheng Yang, Zhaoying Liao, Ling Xu, Conghua Li, Huan Zeng, Jinlin Song, Lei Zhang

**Affiliations:** 1 College of Stomatology, Chongqing Medical University, Chongqing, China; 2 Chongqing key Laboratory of Oral Diseases and Biomedical Sciences, Chongqing, China; 3 Chongqing Municipal Key Laboratory of Oral Biomedical Engineering of Higher Education, Chongqing, China; 4 Children’s Hospital of Chongqing Medical University, Chongqing, China; 5 School of Public Health and Management, Chongqing Medical University, Chongqing, China; 6 Research Center for Medicine and Social Development, Chongqing Medical University, Chongqing, China; 7 The Innovation Center for Social Risk Governance in Health, Chongqing Medical University, Chongqing, China; 8 Melbourne Sexual Health Centre, Alfred Health, Melbourne, VIC, Australia; 9 Central Clinical School, Faculty of Medicine, Nursing and Health Sciences, Monash University, Melbourne, VIC, Australia; 10 Research Center for Public Health, School of Medicine, Tsinghua University, Beijing, 100084, China

## Abstract

Early childhood caries (ECC) is the most common chronic disease in young children. Its reported prevalence varies greatly across China. This systematic review aimed to explore the epidemiological characteristics of ECC in mainland China from 1987 to 2013. In total, 102 articles were included. The pooled national prevalence and care index (ft/dmft%) for ECC were 65.5% and 3.6%, respectively. The overall ECC prevalence declined from 77.9% during 1987–1994 to 56.4% during 2010–2013. The pooled ECC prevalence for children aged 1–6 years was 0.3%, 17.3%, 40.2%, 54.4%, 66.1%, and 70.7%, respectively. There was no significant difference in prevalence between boys (59.1%) and girls (58.9%); and the care index was also similar (8.1% versus 7.7%). Slightly higher ECC prevalence was observed in rural areas (63.5%) compared with urban areas (59.5%) (RR = 1.08, 95% CI: 1.02–1.14); but a much higher care index was reported in urban children (6.0%) than their rural counterparts (1.6%) (RR = 3.68, 95% CI: 2.54–5.35). The 2006–2013 map of ECC prevalence among 5-year-olds showed wide geographic variations across China. Four adjacent provinces, including Sichuan, Chongqing, Hubei, and Shaanxi, constituted the areas with the lowest ECC prevalence in mainland China.

Dental caries, progressive damage to teeth caused by bacteria, is a widespread chronic disease that affects 60–90% of school-aged children and the vast majority of adults in most industrialized countries^1^. A review published in 2009 indicated an alarming increase in the global prevalence of dental caries affecting children as well as adults^2^. Early childhood caries (ECC) is defined as the presence of one or more decayed (noncavitated or cavitated lesions), missing (due to caries) or filled tooth surfaces in any primary (deciduous) tooth in a child aged 6 years or younger^3^. It has been reported as the most prevalent infectious paediatric disease, and it is five, four and twenty times more common than asthma, early childhood obesity and diabetes, respectively^4^. If untreated, ECC causes pain and infection, which may not only interfere with a child’s correct chewing movement and nutrition intake, but may also impact on the development of permanent dentition and general heath^1^,^4^,^5^. Furthermore, oral diseases affect children’s attendance at school, contributing to more than 51 million school hours lost in the US in 2000 alone^4^. The global disability-adjusted life-years (DALYs) due to untreated deciduous caries increased from 405,000 in 1990 to 426,000 in 2010^6^. The classical restorative treatment for deciduous caries is extremely costly and time consuming. Dental caries has become a major public health issue for children worldwide and placed a huge economic burden on families and society.

The disease burden of childhood ECC varies substantially worldwide. In some European countries, ECC is not common, with prevalence ranging from 11.4% among 3–6 year olds in Sweden^7^ to 19.0% among 3–5 year olds in Italy^8^. A high prevalence of ECC has been reported in some Middle Eastern countries, such as Palestinian (76%)^9^ and the United Arab Emirates (83%)^10^. According to the national surveys from several countries, such as Greece (36%)^11^, Brazil (45.8%)^12^, India (51.9%)^13^, and Israel (64.7%)^14^, the prevalence at age 5 years appears to be inconsistent.

China is the most populous and rapidly developing country in the world. The number of children under 6 years of age in China reached 130 million in 2010^15^. For effective intervention and optimal allocation of medical resources in this population, it is essential to understand the current prevalence trends and treatment status of ECC in China. Over the past 30 years, China has experienced rapid socioeconomic changes, with an average annual economic growth rate as high as 9.8%, and the per capita gross domestic product increasing from 1,112 RMB in 1987 to 38,420 RMB in 2012^16^. Such development may have had a remarkable influence on the prevalence of ECC, as the effect of socioeconomic status on ECC has been confirmed in many studies^17^,^18^. More developed Chinese provinces, such as Guangdong^19^,^20^, Fujian^21^ and Hainan^22^, and many other cities in mainland China have conducted epidemiological surveys on primary dental caries. However, their findings have varied greatly. The reported prevalence of ECC ranges from 14.9% in Shaanxi^23^ to 87.3% in Heilongjiang^24^. The level of treatment, assessed by the care index, which refers to the percentage of teeth treated by filling relative to the total number of teeth that are decayed, missing or filled (ft/dmft%), also varies considerably from 0.2% in Hebei^25^ to 49.8% in Beijing^26^. Two national oral health surveys conducted in 1995 and 2005 reported that the dental caries rate in 5-year-old children was 76.5% and 66.0%, respectively^27^,^28^. There have not been any further national surveys on ECC carried out in mainland China since then.

To date, there are no systematic reviews published on ECC prevalence and care index in mainland China. In particular, some important questions still remain unanswered: what is the overall prevalence and treatment level for ECC in mainland China? Are there temporal and spatial distribution trends? Is there a correlation between ECC prevalence with age, gender and location of residence (e.g. urban versus rural)? To advance our understanding of these issues, it is necessary to use all of the available evidence from China to explore the epidemiological characteristics of this disease over the past30 years. Hence, we conducted a systematic review of the published literatures on the prevalence and treatment status of dental caries among children aged 1–6 years in mainland China (excluding Hong Kong, Taiwan, and Macao). The aim of this evaluation was to provide evidence to inform health programme planning and policy-making for caries prevention and treatment among children.

## Results

### Literature search and quality assessment

**Figure 1:**
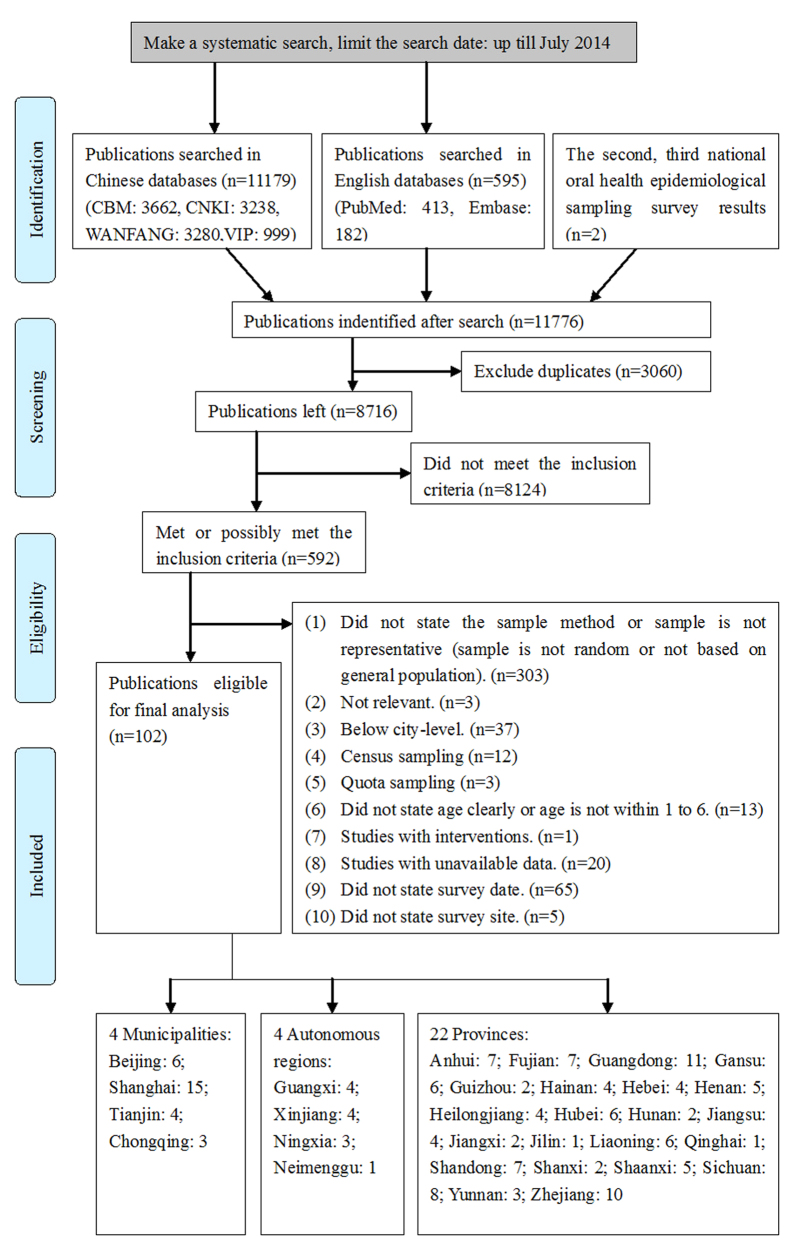
Flow chart of literature search and selection.

A total of 11,776 publications were identified, and 102 eligible articles were included in the meta-analysis; of which, 100 were written in Chinese and two in English (Fig. 1). There were two national-level, 20 provincial-level, and 80 city-level articles, which involved 22 provinces, four municipalities, and four autonomous regions. The total sample size was 349,215. The characteristics of the 102 articles were summarized in Supplementary Table S1. Of these studies, 91 used the diagnostic criteria for caries defined by the World Health Organization (WHO)^29^ or the Chinese National Epidemiological Survey Programs of Oral Health^27^,^28^. The criteria include obvious cavities, sub-face enamel lesions, demineralization of dentine or localized enamel breakdown or noncavitation enamel damage on pits and fissures, and smooth surface (contacting surface, facial, and lingual surface), or definite soft cave bottom or tunnel wall. All of the surveys were conducted in preschools with visual–tactile or visual only methodology. In 77 studies, the examiners were trained dentists, medical doctors or medical students. The qualifications of examiners were not reported in 25 of the studies. The examiners were calibrated with an inter-rater consistency test (Kappa value >0.85) in 45 of the studies. Quality assessment showed that all of the studies scored at least 7 out of 10 and the numbers of studies scored from 7 to 10 were 10, 71, 15 and 6 respectively (Supplementary Table S2 on line).

### Prevalence of ECC in mainland China

#### ECC prevalence over time

**Figure 2:**
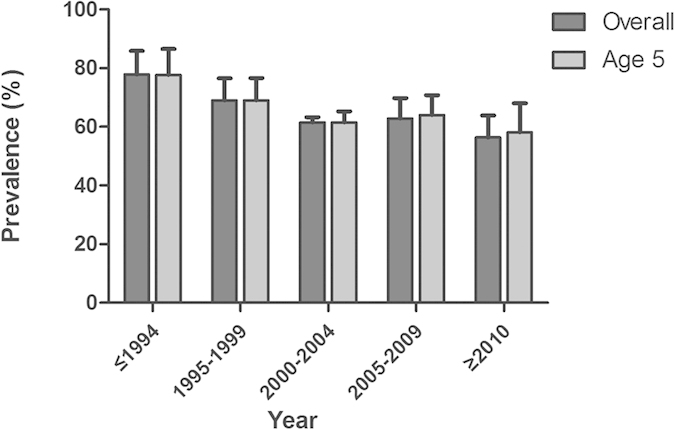
Temporal trend of early childhood caries prevalence in mainland China during 1987–2013.

The pooled overall prevalence of ECC in mainland China was 65.5% (95% CI: 58.6–71.9%, Table 1). A total of 102 studies reported the prevalence of ECC during 1987–2013, while 76 studies conducted reported ECC prevalence at age 5. The overall prevalence of ECC ranged from 77.9% in 1987–1994 to 56.4% in 2010–2013, while the prevalence at age 5 ranged from 77.7% in 1987–1994 to 58.1% in 2010–2013. Both indicators revealed a substantial declining trend over time (Fig. 2).

#### ECC prevalence by age

**Figure 3:**
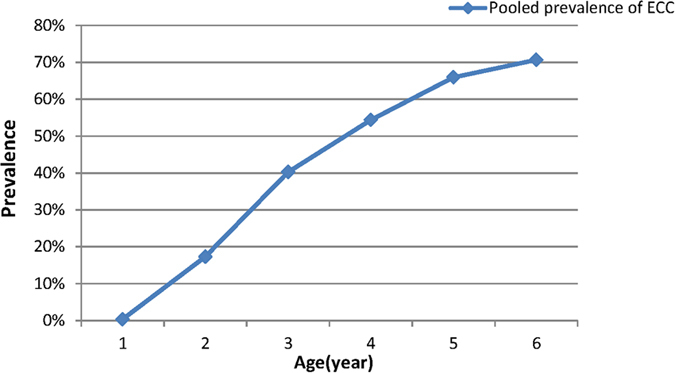
Early childhood caries prevalence of different age group in mainland China during 1987–2013.

The pooled results of 91 articles indicated that the overall prevalence of ECC for children aged 1–6 years was 0.3%, 17.3%, 40.2%, 54.4%, 66.1% and 70.7%, respectively (Table 1), showing an increasing trend with age (Fig. 3). Specifically, the estimated prevalence of ECC among 5-year-olds was 66.1% (95% CI: 59.0–73.4%, Table 2).

#### ECC prevalence by gender

A total of 72 articles reported the ECC prevalence of boys and girls aged 1–6 years separately. The prevalence of ECC in boys and girls was 59.1% (95% CI: 55.0–63.1%) and 58.9% (95% CI: 54.9–62.9%), respectively. The gender difference was not statistically significant (RR = 1.00, 95% CI: 0.99–1.02). In the 31 studies that stratified prevalence of ECC in 5-year-olds by gender, the prevalence for boys and girls aged 5 years was 67.5% (95% CI: 64.3–70.8%) and 68.7% (95% CI: 65.4–72.0%), respectively , and the difference was not statistically significant (RR = 1.00, 95% CI: 0.99–1.01).

#### ECC prevalence by area

**Figure 4:**
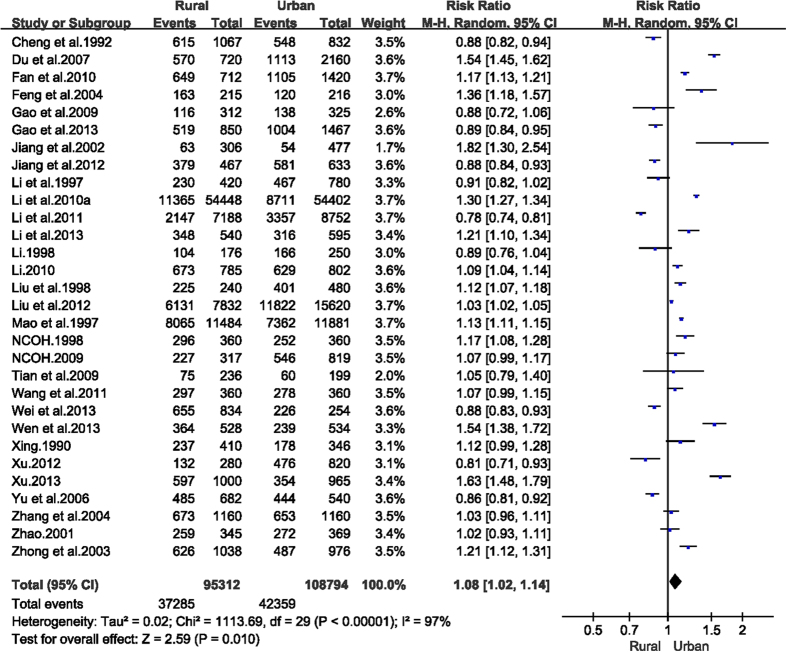
Forest plot of early childhood caries prevalence in rural and urban areas of mainland China during 1987–2013.

A total of 30 articles reported ECC prevalence in children aged 1–6 years from both urban and rural areas. The pooled prevalence of ECC in rural and urban China was 63.5% (95% CI: 52.1–75.0%) and 59.5% (95% CI: 47.8–71.2%), respectively. The RR for rural versus urban areas was 1.08 (95% CI: 1.02–1.14, Fig. 4). Only 19 articles reported the prevalence of ECC at age 5 by residential areas. In rural China, the prevalence of ECC at age 5 was 68.2% (95% CI: 62.2–74.3%), in comparison with 63.3% in the urban areas (95% CI: 56.7–69.8%). Again, the RR of developing dental caries was significantly higher in rural China (RR = 1.08, 95% CI: 1.03–1.14).

#### Geographical distribution of ECC prevalence

**Figure 5:**
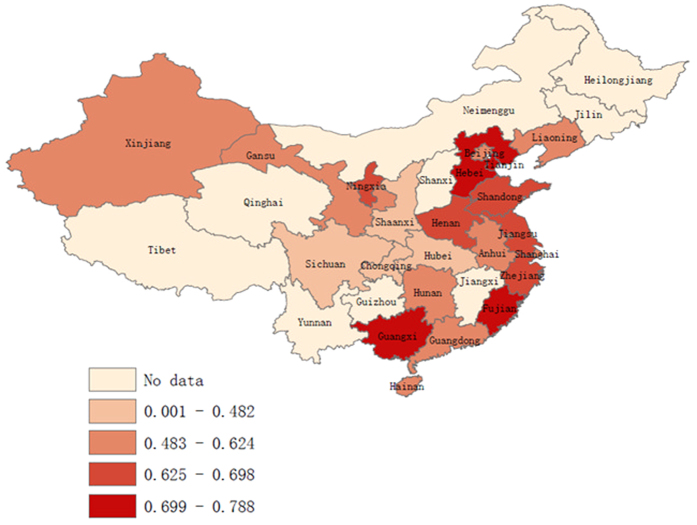
Spatial distribution of early childhood caries prevalence at age 5 in mainland China during 2006–2013 (created by the ArcGIS software).

The prevalence of ECC in 5-year-old children from different provinces in mainland China from 2006 to present was combined. The data were reported in 47 articles, covering 22 provinces (data were not available in 9 provinces: Guizhou, Heilongjiang, Jiangxi, Jilin, Qinghai, Shanxi, Yunnan, Neimenggu and Tibet). Five distribution zones on the map with different colours were created based on the prevalence of ECC (Fig. 5). The geographical distribution of ECC showed substantial variations across provinces. The lowest prevalence zone appeared on the map in light red, and was observed in the middle and western regions of China in four adjacent provinces: Chongqing (36.4%), Sichuan (38.9%), Hubei (40.9%), and Shaanxi (48.2%). The highest prevalence, shown on the map in the darkest red, was observed for Guangxi (78.8%), Hebei (78.8%), Tianjin (76.2%), and Fujian (73.7%).

### Care index of ECC in mainland China

#### Care index over time

**Figure 6:**
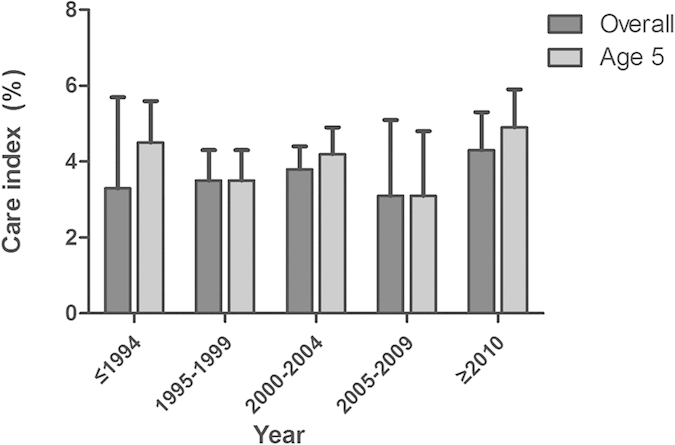
Temporal trend of care index of early childhood caries in mainland China during 1987–2013.

A total of 44 studies reported the overall care index for ECC from 1988 to 2012. The pooled overall care index for ECC was 3.6% (95% CI: 2.6–5.0%, Table1). The lowest overall care index was 3.1% estimated in 2005–2009, and the highest was 4.3% in 2010–2013. The care index at age 5 was reported in 29 studies. There appeared to be an increase in care index from 3.1% in 2005–2009 to 4.9% in 2010–2013. However, the temporal trend was not significant due to insufficient information (Fig. 6).

#### Care index by age

The pooled national care index for ECC for children aged 3–6 years was 2.8%, 4.0%, 4.0%, and 4.3%, respectively. There were no data reported for children under age 2 (Table 1). Specifically, 29 articles described the care index for ECC at age 5, the pooled estimate was 4.0% (95% CI: 3.0–5.1%, Table 2).

#### Care index by gender

**Figure 7:**
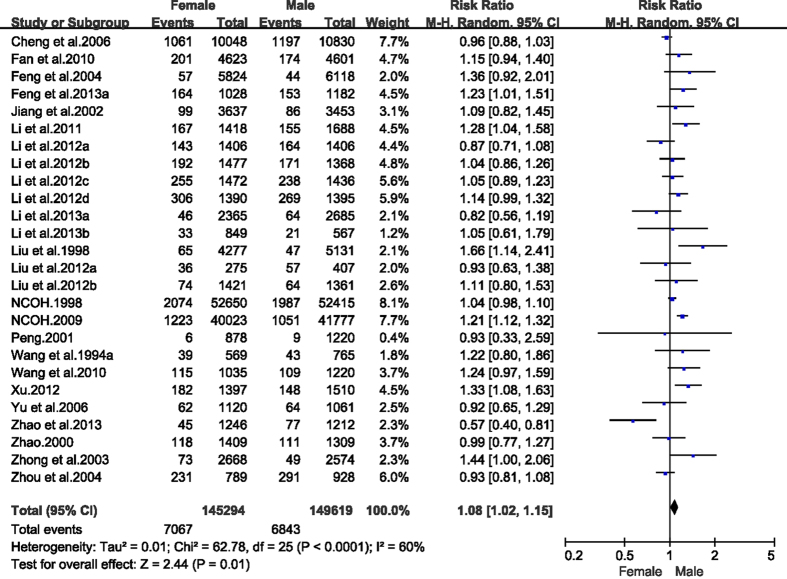
Forest plot of care index of early childhood caries among different gender in mainland China during 1987–2013.

A total of 23 articles reported the care index for ECC for 3–6 year olds in both genders. The pooled care index was 7.7% for boys (95% CI: 6.6–8.7%) and 8.1% for girls (95% CI: 7.0–9.2%). Statistically, the likelihood of receiving care was higher in girls than in boys (RR = 1.08, 95% CI: 1.02–1.15, Fig. 7). Data on the care index stratified by gender at age 5 were reported in 21 articles. The corresponding care index for boys and girls was 9.8% (95% CI: 8.5–11.2%) and 10.0% (95% CI: 8.6–11.5%), respectively.

#### Care index by area

**Figure 8:**
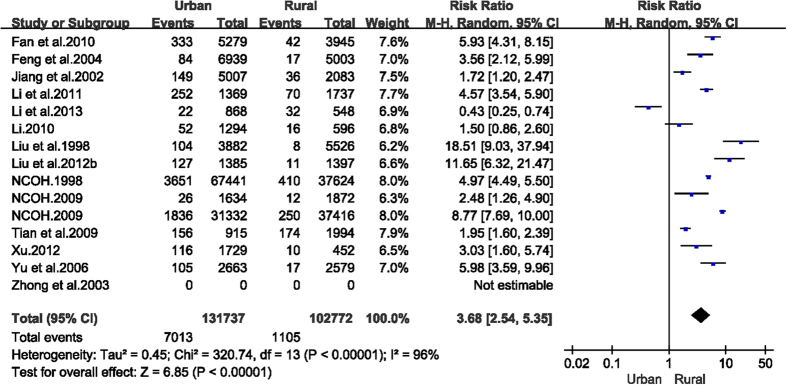
Forest plot of care index of early childhood caries among rural and urban areas of mainland China during 1987–2013.

Data were pooled from 14 articles to estimate the care index for ECC in 3–6 year olds by urban and rural area. Urban children were much more likely to receive care than their rural counterparts (6.0%, [95% CI: 4.7–7.3%] versus 1.6% [95% CI: 1.2–1.9%], RR = 3.68 [95% CI: 2.54–5.35], Fig. 8). Similarly, estimated care index for children at age 5 was significantly higher in urban children (5.1%, 95% CI: 3.6–6.6%) than rural children (1.2%, 95% CI: 0.9–1.6%) (RR = 3.27, 95% CI: 2.20–4.88).

#### Publication bias

Publication bias was observed across the studies that reported ECC prevalence and care index. The shape of the funnel plots was skewed above, suggesting the existence of publication bias may overestimate the actual rates (Supplementary Fig. S1 online). Publication bias for both rates were statistical significant (Begg’s test, P < 0.001).

## Discussion

Oral health in children is an important public issue in China and worldwide. This study is the first published systematic review on the prevalence and care index of ECC in mainland China. The results presented here summarize the epidemiology of caries over the last 30 years among Chinese children, and provide substantial evidence for clinical practice and further research.

Pooled-summary for the included studies highlights that caries among Chinese children remains a serious and urgent problem, similar to findings in other developing countries^30^. The pooled prevalence of ECC was relatively high (65.5% overall and 66.1% at age 5), while the care index was extremely low (3.6% overall and 4.0% at age 5). Tooth decay was traditionally regarded as a disease prevalent in developed countries, but less prevalent in developing countries^31^. However, this trend has been reversed recently, largely due to the dietary and lifestyle changes globally^32^,^33^. According to the two national health and nutrition surveys conducted in the United States in 1988–1994 and 1999–2004^34^, the prevalence of primary dental caries in 2–5-year-olds was 24.23% and 27.90%, respectively, and the respective values for the care index were approximately 33.66% and 40.17%. In England in 2005, 39.6% of 5-year-olds were reported to have obvious dental caries and approximately 11% of decayed teeth were treated with filling^35^. The prevalence of ECC reported in some developed countries in Europe is less than 20%^7^,^8^,^36^. In contrast, higher prevalence is observed in low-income countries. In the Philippines, national oral health surveys have reported high prevalence of ECC among 5–6-year-old children (97% in 1982, 95% in 1987, 95% in 1992, and 94% in 1998), while the care index was only 1% or less^37^. In addition, our results indicate that dental caries are prevalent even among very young children in mainland China. For children aged 3 years old, 40.2% had dental caries, most of which were untreated, compared to only 8.7% of their contemporaries in Italy^8^. Also, oral health of children under 3 years old should not be neglected.

This study demonstrates a declining trend in the prevalence of ECC over the past 30 years, although it is still far from the target set by the WHO in 2000 for 50% of children at age 6 to be caries-free^38^. This study indicates that the oral health status of children in China has improved over time, most likely due to the improvements in public health services and improved awareness about oral health. For example, governmental health expenditure has increased from 51.9 billion RMB in 1980 to 1778.9 billion RMB in 2006, and the number of dentists has grown rapidly from 11,044 in 1985 to 136,520 in 2008^30^,^39^. Furthermore, extensive health education programmes, such as the annual Love Teeth Day (LTD) campaign that has been implemented every September since 1989, have been organized to promote individual oral health across the country^31^. Parental knowledge and attitudes toward oral health have also improved according to two national surveys conducted in 1995 and 2005^27^,^28^. However, challenges still remain compared with developed countries. Firstly, although the number of dentist has increased, the dentist to population ratio is only 1:10,000 in mainland China, far below the average of approximately 1:2,000 in most developed countries. Secondly, over 85% of the total oral health expenditure is not covered by health insurance^30^. Thirdly, there is a general misunderstanding that primary teeth are temporary and may not be necessary for treatment. All of which may contribute to the low levels of treatment observed. Therefore, there is an urgent need to scale-up prevention and treatment of ECC in young children in mainland China.

Previous studies^11^,^40^,^41^ reported that rural children had higher risks of caries compared with their urban counterparts. However, with the rapid economic development and improvement of health awareness in rural areas, the difference between urban and rural areas has narrowed recently^42^; and our study did not identify significant difference in prevalence of ECC in urban and rural China (63.5% vs 59.5%). However, we should acknowledge the possibility of discrepancy in the two areas as health institutes in urban China do appear to be more resourceful and systematically conduct more epidemiologic surveys of ECC than their rural counterparts. Further investigations are warranted to elucidate this geographical difference. Notably, a much lower care index was found in rural areas than in urban. Unequal distribution of oral health services and resources between urban and rural areas may account for the better dental healthcare seen in children living in urban areas in mainland China. In 2005, the average healthcare expenditure per person was 1,248 RMB in urban areas compared to only 362 RMB in rural areas^30^. The number of dentists in urban areas is 4–5 times of that in rural areas^39^,^43^. Therefore, the government and healthcare planners should focus more on the treatment of ECC in rural areas.

The pooled results of the included studies showed non-significant differences in ECC prevalence between genders, which was consistent with some previous studies^8^,^9^,^44^, but contradictory to others^45^,^46^. According to a meta-analysis of the gender difference in oral health among 5-6-year-olds in South Asia, 3 of 5 ethnic groups showed significantly higher caries prevalence among boys, while 2 groups showed no significant gender difference^47^. Previous studies have seldom compared the difference in treatment levels between boys and girls. Our meta-analysis of data from 23 relevant studies indicated that the combined care index was similar in girls and boys, but the results require further exploration to confirm its validity.

The latest national epidemiological survey on ECC in China was conducted in 2006, and the geographical distribution of ECC has been unclear for almost 10 years. In this study, we used a geographic information system to present the geographical distribution of the ECC prevalence for mainland China from 2006 to 2013. The results showed wide variations across the country. Considering China covers a vast territory with unbalanced economic development, these variations may be caused by latitude, socioeconomic status, or cultural differences. This prevalence map could benefit the future allocation of oral medical resources in mainland China. Interestingly, four neighbouring provinces in the middle and western region of China, including Chongqing (36.4%), Sichuan (38.9%), Hubei (40.9%) and Shaanxi (48.2%), constituted an area of low ECC prevalence. Potential reasons may include a traditional lifestyle and the dietary habits prevailing in these regions^31^. However, further research is required to confirm this assumption.

This study has several limitations. First, heterogeneity is unavoidable in a meta-analysis, especially in a meta-analysis of cross-sectional studies^48^. We followed strict inclusion and exclusion criteria (only random-sampling surveys were adopted) to minimize the influences of different survey methods and designs. However, several factors may still have potential effects on the heterogeneity in this study: (1) the surveys were carried out at different sites and at various times with different socioeconomic conditions; (2) large differences existed in sample sizes in the included studies; (3) although subgroup analyses were conducted, heterogeneity within each subgroup was still significant as it may also be influenced by other factors including obesity, ethnic differences, eating habits, and parental attitudes, which we did not have sufficient data to account for; (4) although the criteria used to diagnose ECC have been comparatively standardized, bias could not be avoided since the diagnosis of caries was mainly dependent on the investigators’ assessment by visual-tactile or visual methods, for instance, identification of interproximal caries is difficult without an X-ray in an epidemiological investigation, which will underestimate the prevalence. More accurate measurements to detect caries, especially noncavitated lesions, need to be advocated in further research; (5) we used the care index (ft/dmft%) to reflect the treatment status. The assessment of missing teeth due to caries is complicated by natural exfoliation of teeth among 5 and 6 years old children. Second, although we weighted estimates according to the target population size in each province, this study covered more urban than rural areas, which may not reflect the actual population structure of China. The country-level estimate may be potentially biased. Third, publication bias across the studies may affect the overall estimates, in this case overestimating them. The bias may due to our selection criteria that only included peer-reviewed articles, but not other publication types. Institutes and hospitals in developed regions may conduct epidemiologic surveys of ECC and publish their studies more frequently, given their available resources. Fourth, some studies only recruited children at a certain age, while others covered children of a wider age range. The difference in age structure could affect the results since the prevalence of ECC increases with age. Therefore, we specifically focused on 5-year-olds for comparisons across studies.

In conclusion, this systematic review presents important epidemiological characteristics of the oral health status among children in mainland China over the last 30 years. Our analysis reports a declining trend of ECC over time. However, the prevalence of ECC is still at a relatively high level, while the care index is extremely low. This study provides important evidence for effective interventions to improve the prevention and treatment of ECC in Chinese children.

## Methods

This study was conducted according to the preferred reporting items for systematic review and meta-analyses (PRISMA) checklist (Supplementary checklist S1 online).

### Search strategy

A comprehensive search strategy was first developed by a research team owning experts on health informatics, dentistry, clinical medicine, epidemiology and statistics; then a pilot search was conducted by the first and second author independently to test the strategy. The final search strategy was subsequently confirmed based on the pilot and group discussion. Peer-reviewed articles were searched in the following English and Chinese databases from the date of establishment to July, 2014: PubMed (1966-), Embase (1974-), Chinese Biomedical Literature Database (CBM) (1978-), Chinese National Knowledge Infrastructure database (CNKI) (1979-), Chinese Wan Fang database (1990-), and Chongqing VIP database (1989-), using the key terms ‘caries’, ‘prevalence’, ‘epidemiology’, and ‘China’. Additionally, a manual search was applied to the reference lists of all of the eligible articles.

### Selection criteria

Studies were included if they (1) were conducted in mainland China (except for Hong Kong, Taiwan, and Macao); (2) were cross-sectional surveys using random sampling; (3) were based on children under 6 years old from the general population rather than a specific group; (4) were conducted at city-level or above; (5) reported sufficient information on the prevalence or care index of ECC. Care index is defined as the proportion of filled teeth to the total number of teeth that are decayed, missing or filled (ft/dmft%)^35^,^49^ to reflect the treatment status; and (6) were written in English or Chinese. Articles were excluded if they (1) recruited children older than 6 years; (2) did not provide details on the survey period or location; (3) used census or quota sampling; and (4) were abstracts, conference proceedings, commentaries, review articles or intervention studies.

### Quality assessment

A tool adopting from the Reporting of Observational Studies in Epidemiology (STROBE) guideline^50^ (Supplementary Table S3 on line) was used to assess the quality of the selected studies. Each bias type was evaluated for individual study (low risk = 2, moderate risk = 1, and high risk = 0) and the total score represented the quality score of bias risk. The maximum score of 10 represents the lowest risk of bias. The assessment was judged by two independent authors, and a final decision was reached by consensus or by the third author when necessary.

### Data extraction

Two authors screened articles and extracted data independently. Any disagreement was resolved by consensus or the third author. We contacted the corresponding authors for further or missing information when necessary. The following information was extracted from each eligible study: (1) publication details including author(s) and year of publication; (2) design of study, including study location and period, sampling method, sample size, and characteristics of the participants; (3) details of targeted indicators, such as diagnosis criteria, examiners, number of cases, number of decayed, missing and filled teeth (dmft), and number of filled teeth (ft), which were subgrouped by year, age, gender, urban/rural area, and province where available.

### Statistical analysis

Meta-analyses were performed by Cochrane Review Manager (RevMan) 5.1and STATA software 11.1 (Stata, College Station, TX, USA). The pooled estimates and 95% confidence intervals for each indicator were calculated by pooling the data from each study. Statistical heterogeneity was detected by Q-test and I^2^-statistics. A random effects model was adopted in the case of significant heterogeneity (I^2^ > 50% or P < 0.1); otherwise, a fixed-effects model was used. If high heterogeneity was observed, subgroup analysis was conducted to explore possible factors including survey year, age distribution, gender, location, and province. The discrepancies between boys and girls, and urban and rural areas were compared using the RR and 95% CI. To reflect spatial distribution of ECC, pooled prevalence estimates for ECC in 5-year-old children in each province during 2006–2013 were entered into the ArcGIS software 10.0 to form a prevalence map. Potential publication bias was assessed by funnel plots and Begg’s test; the result was considered to be significant if P ≤ 0.05.

To obtain country-level estimate, we employed a qualified approach that weights prevalence estimates in each province according to the number of children present in the provinces^15^. Briefly, based on data availability, studies were categorized into five time periods of data collection: ≤1994, 1995–1999, 2000–2004, 2005–2009, ≥2010. Then, for each province, subgroup meta-analysis was conducted to obtain an estimate for each of these time periods for each outcome indicator. In each time period, the national ECC prevalence and care index was estimated as the average prevalence levels across all the provinces weighted by the population size of children under six years old in each province (e.g. provincial estimates from more populous provinces have greater weight). This approach has already been published in past literature by Zhang, L. *et al.*^51^,^52^.

## Additional Information

**How to cite this article**: Zhang, X. *et al.* Prevalence and care index of early childhood caries in mainland China: evidence from epidemiological surveys during 1987–2013. *Sci. Rep.* **6**, 18897; doi: 10.1038/srep18897 (2016).

## Supplementary Material

Supplementary Information

## Figures and Tables

**Table 1:** Pooled prevalence and care index of early childhood caries in mainland China during 1987–2013.

	Number of study	sample size	case	Pooled prevalence (%)	95%CI (%)
Overall prevalence	102	349,215	154,476	65.5	58.6–71.9
*Time period*					
* 1987*–*1994*	15	16,456	11,933	77.9	69.2–85.9
* 1995*–*1999*	21	174,327	58,004	69.0	61.4–76.5
* 2000*–*2004*	14	21,024	11,428	61.5	57.7–63.3
* 2005*–*2009*	34	100,332	54,115	62.9	56.0–69.8
* 2010*–	25	37,076	18,996	56.4	48.8–63.9
Age					
* 1*	4	32,605	412	0.3	0–0.7
* 2*	15	21,011	2,563	17.3	12.0–22.6
* 3*	56	15,185	43,506	40.2	27.2–53.3
* 4*	55	65,077	32,377	54.4	42.3–66.5
* 5*	76	121,010	78,009	66.1	59.0–73.4
* 6*	42	24,283	15,299	70.7	57.4–84.0
	**Number of study**	**dmft**	**ft**	**Pooled care index (%)**	**95% CI (%)**
Overall care index	44	405,040	21,441	3.6	2.6–5.0
*Time period*					
* 1987*–*1994*	6	21,045	1,348	3.3	2.7–5.7
* 1995*–*1999*	10	149,321	5,192	3.5	2.6–4.3
* 2000*–*2004*	7	29,841	1,124	3.8	3.1–4.4
* 2005*–*2009*	13	149,042	7,847	3.1	1.1–5.1
* 2010-*	12	55,791	5,930	4.3	3.3–5.3
Age					
* 1*	/	/	/	/	/
* 2*	/	/	/	/	/
* 3*	13	21,246	1,115	2.8	1.3–4.4
* 4*	10	24,329	2,305	4.0	1.8–6.2
* 5*	29	291,181	14,695	4.0	3.0–5.1
* 6*	12	30,267	1,929	4.3	2.5–6.2

ECC: early childhood caries; ft: number of filled teeth; dmft: number of decayed, missing and filled teeth; care index: ft/dmft; CI: confidence interval.

**Table 2:** Pooled prevalence and care index of early childhood caries at age 5 in mainland China during 1987–2013.

	Number of study	Sample size	Case	Pooled prevalence (%)	95% CI (%)
Prevalence	76	121,010	78,009	66.1	59.0–73.4
*Time period*					
* 1987*–*1994*	11	3,648	2,802	77.7	68.9–86.5
* 1995*–*1999*	15	48,978	31,645	69.0	61.4–76.6
* 2000*–*2004*	12	12,507	7,750	61.5	57.7–65.3
* 2005*–*2009*	28	43,843	28,372	64.0	57.0–70.8
* 2010-*	14	12,034	7,440	58.1	50.2–68.0
	**Number of study**	**dmft**	**ft**	**Pooled care index (%)**	**95% CI (%)**
Care index	29	293,362	14,821	4.0	3.0–5.1
*Time period*					
* 1987*–*1994*	3	4,613	35	4.5	3.3–5.6
* 1995*–*1999*	7	123,459	4,641	3.5	2.8–4.3
* 2000*–*2004*	6	28,914	1,107	4.2	3.6–4.9
* 2005*–*2009*	10	114,672	5,246	3.1	1.4–4.8
* 2010*–	5	21,704	3,475	4.9	3.8–5.9

ECC: early childhood caries; ft: number of filled teeth; dmft: number of decayed, missing and filled teeth; care index: ft/dmft%; CI: confidence interval.
