# Supplementary material for: Prevalence and care index of early childhood caries in mainland China: evidence from epidemiological surveys during 1987–2013
Source: Sci Rep. 2016 Jan 13;6:18897. doi: 10.1038/srep18897 (PMC4725361; doi:10.1038/srep18897)
Supplement: Supplementary Information [file srep18897-s1.pdf]

Prevalence and care index of early childhood caries in mainland China: evidence from epidemiological surveys during 1987–2013

Xiaonan Zhang, Sheng Yang, Zhaoying Liao, Ling Xu, Conghua Li, Huan Zeng, Jinlin Song, Lei Zhang

Corresponding to:

HuanZeng, School of Public Health and Management, Chongqing Medical University, Chongqing, China, No. 1 Yixueyuan Road, Yuzhong District, Chongqing, China; Tel: +86 23 68485008; Fax: +86 23 68485008; E-mail:zenghuan586@aliyun.com

Jinlin Song, College of Stomatology, Chongqing Medical University, Chongqing, China, No.426 Songshibei Road, Yubei District, Chongqing, China; Tel: +86 23 89035721; Fax: +86 23 89035721; E-mail: dentistcq@163.com

Table S1 | Characteristic of the included 102 studies

First author & published year	Survey date	Provinces	Territorial levels	U&R	Investigators	Sampling method	Diagnostic criteria	Sample size	Case size	Prevalence (%)	dmft	ft	Care index (%)	Quality score
**Cai et al.2013**[1]	2011	Shanghai	city	U	dentists	cluster	Guideline for the 3rd National Oral Health Survey	1259	463	36.78	1640	_	_	8
**Chen et al.2008**[2]	2007	Hainan	city	U	experienced child health doctors	random	WHO oral health survey	2354	1443	61.3	4762	_	_	8
**Chen.2009**[3]	2007	Hainan	city	U	experienced child health doctors	random	WHO oral health survey	1866	1051	56.32	5048	_	_	8
**Cheng et al. 2011**[4]	2009	Anhui	city	U	NA	stratified cluster	<Child Health care> 3rd edition	2993	1418	47.38	_	_	_	7
**Chen et al.1992**[5]	1990	Guangdong	provincial	U&R	health care researchers and medical students	multistage random	WHO oral health survey	1899	1163	61.24	11430	_	_	9
**Cheng et al.2006**[6]	2005	Liaoning	city	NA	trained experienced dentists	random	Guideline for the 2nd National Oral Health Survey	5375	3832	71.29	20878	2258	10.82	9
**Chen et al.2008**[7]	2006	Zhejiang	city	U	experienced doctors	cluster random	WHO oral health survey	1160	995	85.78	7158	_	_	8
**Deng et al.1998**[8]	1995	Hunan	city	U	health care doctors	cluster random	NA	2956	1569	53.08	7175	248	3.46	8
**Du et al.2007**[9]	2001	Guangxi, Hubei	provincial	U&R	trained experienced dentists	stratified random	WHO oral health survey	2014	1113	55.26	5178	_	_	9
**Fan et al.2010**[10]	2008	Guangdong	city	U&R	NA	multi-stage stratified random	Guideline for the 3rd National Oral Health Survey	2880	1683	58.44	9224	375	4.07	10

**Feng et al.2004**[11]	2000	Fujian	provincial	U&R	trained dental students	stratified cluster	WHO oral health survey	2132	1754	82.27	11942	101	0.85	9
**Feng et al.2013(1)**[12]	2011	Shanghai	city	NA	dentists	cluster	WHO oral health survey	1015	526	51.82	2210	317	14.34	8
**Feng et al.2013(2)**	2003	Shanghai	city	NA	dentists	cluster	WHO oral health survey	812	530	65.27	_	_	_	8
**Gao et al.2009a**[13]	2007	Anhui	provincial	U&R	dentists	multi-stage stratified random	WHO oral health survey	431	283	65.66	1463	_	_	9
**Gao et al.2009b**[14]	2008	Hebei	city	R	dentists	cluster random	WHO oral health survey	2040	1002	49.12	4771	9	0.19	8
**Gao et al.2010**[15]	2008	Xinjiang	city	U	trained doctors	stratified cluster random	Guideline for the 2nd National Oral Health Survey	1483	602	40.59	_	_	_	8
**Dong et al.2013**[16]	2012	Shandong	city	U&R	NA	cluster	National uniform standards of dental caries	637	254	39.87	_	_	_	8
**Gou et al.2010**[17]	2008	Chongqing	city	U	NA	cluster random	WHO oral health survey	3804	1030	27.08	13543	_	_	8
**Hu et al.1991**[18]	1989	Henan	city	NA	NA	cluster	WHO oral health survey	207	144	69.57	572	_	_	8
**Hu et al.2008**[19]	2007	Jiangsu	city	U	dentists	random	Guideline for the 2nd National Oral Health Survey	2105	1074	51.02	6049	755	12.48	7
**Hu.2013**[20]	2012	Anhui	city	U	dentists	random	WHO oral health survey	3497	1413	40.41	6010	123	2.05	8
**Jiang et al.2002**[21]	1999	Anhui	provincial	U&R	NA	multi-stage stratified random	Guideline for the 2nd National Oral Health Survey	2317	1523	65.73	7090	185	2.61	10
**Jiang et al.2012**[22]	2011	Shaanxi	city	U	trained pediatricians	cluster	WHO oral health survey	783	117	14.94	_	_	_	8

**Le et al.1992**[23]	1992	Shanghai	city	NA	trained investigators	random	WHO oral health survey	1112	889	79.95	5103	306	6	8
**Li et al.1991**[24]	1988	Hubei	city	NA	dentists	cluster	Criteria from <Oral Medicine>	924	702	75.97	3825	_	_	8
**Li et al.1997**[25]	1995	mainland	China	U&R	pediatricians	cluster random	WHO oral health survey	108850	20076	18.44	_	_	_	9
**Li et al.2007**[26]	2006	Fujian	city	NA	NA	random	WHO oral health survey	1760	950	53.98	4076	_	_	8
**Li et al.2010a**[27]	2008	Fujian	city	U&R	dentists	multi-stage stratified random	Guideline for the 3rd National Oral Health Survey	1523	1093	71.77	7157	_	_	8
**Li et al.2010b**[28]	2008	Tianjin	provincial	U&R	NA	stratified cluster random	Guideline for the 3rd National Oral Health Survey	15940	5504	34.53	_	_	_	8
**Li et al.2011**[29]	2009	Shanghai	city	U&R	NA	multi-stage stratified random	WHO oral health survey	1135	664	58.5	3106	322	10.37	8
**Li et al.2012(1)**[30]	2008	Shanghai	provincial	U	dentists	cluster random	Guideline for the 3rd National Oral Health Survey	950	603	63.47	2812	307	10.92	8
**Li et al.2012(2)**	2009	Shanghai	provincial	U	dentists	cluster random	Guideline for the 3rd National Oral Health Survey	950	608	64	2845	363	12.76	8
**Li et al.2012(3)**	2010	Shanghai	provincial	U	dentists	cluster random	Guideline for the 3rd National Oral Health Survey	900	584	64.89	2908	493	16.95	8
**Li et al.2012(4)**	2011	Shanghai	provincial	U	dentists	cluster random	Guideline for the 3rd National Oral Health Survey	900	580	64.44	2785	575	20.65	8
**Li et al.2013a**[31]	2011	Hebei	city	NA	dentists	stratified cluster	WHO oral health survey	1123	761	67.76	5050	110	2.18	8
**Li et al.2013b**[32]	2012	Shandong	city	U&R	experienced dentists	stratified random	Guideline for the 3rd National Oral Health Survey	426	270	63.38	1416	54	3.81	8

**Li.1998[33]**	1996	Heilongjiang	city	U&R	NA	random	NA	1100	960	87.27	_	_	_	7
**Li.2001[34]**	1988	Jiangxi	city	NA	dentists	stratified cluster random	Guideline for the 2nd National Oral Health Survey	561	397	70.77	1661	_	_	8
**Li.2010[35]**	2008	Gansu	city	U&R	health care doctors	stratified cluster random	Guideline for the 3rd National Oral Health Survey	1200	697	58.08	1890	68	3.6	8
**Li.2012[36]**	2011	Tianjin	city	U	specialists from prevention station	random	Guideline for the 3rd National Oral Health Survey	795	524	65.91	2763	_	_	9
**Liang et al.2010[37]**	2009	Guangdong	city	U	dentists and health care doctors	cluster random	Guideline for the 2nd National Oral Health Survey	1763	829	47.02	3836	_	_	7
**Liang et al.2012[38]**	2011	Guangdong	city	U	health care doctors	cluster random	Guideline for the 3rd National Oral Health Survey	4121	2214	53.72	12364	_	_	8
**Lin et al.2003[39]**	2002	Chongqing	provincial	U&R	dentists	stratified cluster	WHO oral health survey	3261	1855	56.88	7411	190	2.56	8
**Lin.2013[40]**	2011	Guangdong	city	U	experienced dentists	multi-stage stratified random	WHO oral health survey	217	107	49.31	543	_	_	8
**Liu et al.1998[41]**	1996	Guangdong	provincial	U&R	dentists	multi-stage cluster random	WHO oral health survey	1587	1302	82.04	9408	112	1.19	9
**Liu et al.2005(1)[42]**	1990	Ningxia	city	U	experienced dentists	random	WHO oral health survey	335	253	75.52	924	89	9.63	8
**Liu et al.2005(2)**	1996	Ningxia	city	U	experienced dentists	random	WHO oral health survey	340	199	58.53	773	77	9.96	8
**Liu et al.2005(3)**	2001	Ningxia	city	U	experienced dentists	random	WHO oral health survey	413	150	36.32	421	46	10.93	8
**Liu et al.2012a[43]**	2012	Ningxia	city	NA	NA	cluster random	WHO oral health survey	225	157	69.78	682	93	13.64	8

**Liu et al.2012b[44]**	2011	Sichuan	provincial	U&R	trained investigators	multi-stage stratified random	Guideline for the 3rd National Oral Health Survey	714	531	74.37	2782	138	4.96	8
**Liu.2009[45]**	2008	Sichuan	city	U	dentists	random	Guideline for the 2nd National Oral Health Survey	1160	408	35.17	1124	_	_	8
**Ma.1997[46]**	1996	Sichuan	city	U	NA	random	NA	4072	1751	43	7037	_	_	8
**Mao et al.1997[47]**	1995	Zhejiang	city	U&R	trained dentists	multi-stage stratified cluster	Guideline for the 2nd National Oral Health Survey	720	626	86.94	4518	24	0.53	9
**Mao et al.2011[48]**	2010	Sichuan	city	R	NA	cluster random	Criteria from <Practical internal medicine >	6024	1710	28.39	_	_	_	7
**Meng et al.2008[49]**	2006	Beijing	city	R	dentists	stratified cluster random	National uniform standards of dental caries	356	145	40.73	487	_	_	8
**NCOH.1998[50]**	1995	Beijing	China	U&R	trained experienced dentists	multi-stage stratified cluster	Guideline for the 2nd National Oral Health Survey	2132	1611	75.56	8911	1002	11.24	10
**NCOH.1998**	1995	Gansu	China	U&R	trained experienced dentists	multi-stage stratified cluster	Guideline for the 2nd National Oral Health Survey	2132	1711	80.25	10532	301	2.86	10
**NCOH.1998**	1995	Guangdong	China	U&R	trained experienced dentists	multi-stage stratified cluster	Guideline for the 2nd National Oral Health Survey	2132	1638	76.83	10255	246	2.4	10
**NCOH.1998**	1995	Hubei	China	U&R	trained experienced dentists	multi-stage stratified cluster	Guideline for the 2nd National Oral Health Survey	2132	1476	69.23	7765	172	2.22	10
**NCOH.1998**	1995	Liaoning	China	U&R	trained experienced dentists	multi-stage stratified cluster	Guideline for the 2nd National Oral Health Survey	2132	1908	89.49	14434	211	1.46	10
**NCOH.1998**	1995	Shandong	China	U&R	trained experienced dentists	multi-stage stratified cluster	Guideline for the 2nd National Oral Health Survey	2132	1607	75.38	8144	118	1.45	10
**NCOH.1998**	1995	Shanghai	China	U&R	trained experienced dentists	multi-stage stratified cluster	Guideline for the 2nd National Oral Health Survey	2132	1669	78.28	10149	895	8.82	10

**NCOH.1998**	1995	Sichuan	China	U&R	trained experienced dentists	multi-stage stratified cluster	Guideline for the 2nd National Oral Health Survey	2132	1292	60.6	5768	420	7.28	10
**NCOH.1998**	1995	Tianjin	China	U&R	trained experienced dentists	multi-stage stratified cluster	Guideline for the 2nd National Oral Health Survey	2132	1590	74.58	7590	128	1.69	10
**NCOH.1998**	1995	Zhejiang	China	U&R	trained experienced dentists	multi-stage stratified cluster	Guideline for the 2nd National Oral Health Survey	2132	1836	86.12	12056	136	1.13	10
**NCOH.1998**	1995	Yunnan	China	U&R	trained experienced dentists	multi-stage stratified cluster	Guideline for the 2nd National Oral Health Survey	2132	1615	75.75	9458	415	4.39	10
**NCOH.2009**[51]	2005	Anhui	China	U&R	trained experienced dentists	multi-stage stratified random	Guideline for the 3rd National Oral Health Survey	795	510	64.15	2572	40	1.56	10
**NCOH.2009**	2005	Beijing	China	U&R	trained experienced dentists	multi-stage stratified random	Guideline for the 3rd National Oral Health Survey	791	464	58.66	2034	364	17.9	10
**NCOH.2009**	2005	Fujian	China	U&R	trained experienced dentists	multi-stage stratified random	Guideline for the 3rd National Oral Health Survey	790	599	75.82	3926	13	0.33	10
**NCOH.2009**	2005	Gansu	China	U&R	trained experienced dentists	multi-stage stratified random	Guideline for the 3rd National Oral Health Survey	785	438	55.8	1899	59	3.11	10
**NCOH.2009**	2005	Guangdong	China	U&R	trained experienced dentists	multi-stage stratified random	Guideline for the 3rd National Oral Health Survey	788	536	68.02	3286	39	1.19	10
**NCOH.2009**	2005	Guangxi	China	U&R	trained experienced dentists	multi-stage stratified random	Guideline for the 3rd National Oral Health Survey	782	652	83.38	4751	25	0.53	10
**NCOH.2009**	2005	Guizhou	China	U&R	trained experienced dentists	multi-stage stratified random	Guideline for the 3rd National Oral Health Survey	767	344	44.85	1465	34	2.32	10
**NCOH.2009**	2005	Hainan	China	U&R	trained experienced dentists	multi-stage stratified random	Guideline for the 3rd National Oral Health Survey	758	577	76.12	3698	38	1.03	10
**NCOH.2009**	2005	Hebei	China	U&R	trained experienced dentists	multi-stage stratified random	Guideline for the 3rd National Oral Health Survey	792	547	69.07	2612	90	3.45	10

**NCOH.2009**	2005	Henan	China	U&R	trained experienced dentists	multi-stage stratified random	Guideline for the 3rd National Oral Health Survey	784	465	59.31	2054	66	3.21	10
**NCOH.2009**	2005	Heilongjiang	China	U&R	trained experienced dentists	multi-stage stratified random	Guideline for the 3rd National Oral Health Survey	789	565	71.61	3195	107	3.35	10
**NCOH.2009**	2005	Hubei	China	U&R	trained experienced dentists	multi-stage stratified random	Guideline for the 3rd National Oral Health Survey	791	448	56.64	1943	31	1.6	10
**NCOH.2009**	2005	Hunan	China	U&R	trained experienced dentists	multi-stage stratified random	Guideline for the 3rd National Oral Health Survey	688	408	59.3	1927	19	0.99	10
**NCOH.2009**	2005	Jiangsu	China	U&R	trained experienced dentists	multi-stage stratified random	Guideline for the 3rd National Oral Health Survey	792	547	69.07	2760	61	2.21	10
**NCOH.2009**	2005	Jiangxi	China	U&R	trained experienced dentists	multi-stage stratified random	Guideline for the 3rd National Oral Health Survey	790	495	62.66	2339	16	0.68	10
**NCOH.2009**	2005	Liaoning	China	U&R	trained experienced dentists	multi-stage stratified random	Guideline for the 3rd National Oral Health Survey	791	585	73.96	3419	75	2.19	10
**NCOH.2009**	2005	Ningxia	China	U&R	trained experienced dentists	multi-stage stratified random	Guideline for the 3rd National Oral Health Survey	744	400	53.76	1551	47	3.03	10
**NCOH.2009**	2005	Shandong	China	U&R	trained experienced dentists	multi-stage stratified random	Guideline for the 3rd National Oral Health Survey	785	524	66.75	2614	112	4.28	10
**NCOH.2009**	2005	Shanxi	China	U&R	trained experienced dentists	multi-stage stratified random	Guideline for the 3rd National Oral Health Survey	792	513	64.77	2589	55	2.12	10
**NCOH.2009**	2005	Shaanxi	China	U&R	trained experienced dentists	multi-stage stratified random	Guideline for the 3rd National Oral Health Survey	787	354	44.98	1833	46	2.51	10
**NCOH.2009**	2005	Shanghai	China	U&R	trained experienced dentists	multi-stage stratified random	Guideline for the 3rd National Oral Health Survey	789	566	71.74	3290	254	7.72	10
**NCOH.2009**	2005	Sichuan	China	U&R	trained experienced dentists	multi-stage stratified random	Guideline for the 3rd National Oral Health Survey	780	458	58.72	2158	50	2.32	10

**NCOH.2009**	2005	Tianjin	China	U&R	trained experienced dentists	multi-stage stratified random	Guideline for the 3rd National Oral Health Survey	761	436	57.29	1853	42	2.27	10
**NCOH.2009**	2005	Xinjiang	China	U&R	trained experienced dentists	multi-stage stratified random	Guideline for the 3rd National Oral Health Survey	788	529	67.13	2561	41	1.6	10
**NCOH.2009**	2005	Zhejiang	China	U&R	trained experienced dentists	multi-stage stratified random	Guideline for the 3rd National Oral Health Survey	788	627	79.57	3813	117	3.07	10
**NCOH.2009**	2005	Chongqing	China	U&R	trained experienced dentists	multi-stage stratified random	Guideline for the 3rd National Oral Health Survey	748	480	64.17	2663	13	0.49	10
**NCOH.2009**	2005	Yunnan	China	U&R	trained experienced dentists	multi-stage stratified random	Guideline for the 3rd National Oral Health Survey	790	504	63.8	2912	57	1.96	10
**NCOH.2009**	2005	Neimenggu	China	U&R	trained experienced dentists	multi-stage stratified random	Guideline for the 3rd National Oral Health Survey	780	576	73.85	3204	172	5.37	10
**NCOH.2009**	2005	Jilin	China	U&R	trained experienced dentists	multi-stage stratified random	Guideline for the 3rd National Oral Health Survey	773	634	82.02	4286	141	3.29	10
**NCOH.2009**	2005	Qinghai	China	U&R	trained experienced dentists	multi-stage stratified random	Guideline for the 3rd National Oral Health Survey	787	562	71.41	2594	50	1.93	10
**Pei.2008**[52]	2007	Henan	city	NA	trained experienced dentists	cluster random	WHO oral health survey	1758	1132	64.39	4683	_	_	8
**Peng et al.2001a**[53]	1997	Guangdong	city	NA	experienced dentists	multi-stage cluster random	NA	639	508	79.5	3093	_	_	8
**Peng et al.2001b**[54]	1998	Zhejiang	city	U	NA	stratified cluster random	WHO oral health survey	832	449	53.97	1761	_	_	7
**Peng.2001**[55]	1998	Zhejiang	city	U	dentists	random	WHO oral health survey	985	529	53.71	2098	15	0.71	8
**Quan.1992**[56]	1990	Hubei	city	U	NA	random	NA	598	329	55.02	1891	_	_	9

**Sheng.2008**[57]	2007	Liaoning	city	NA	dentists	cluster	WHO oral health survey	604	384	63.58	1927	290	15.05	8
**Shu et al.2000**[58]	1996	Shanghai	city	NA	NA	cluster	Guideline for the 2nd National Oral Health Survey	343	144	41.98	772	117	15.16	8
**Song et al.2011**[59]	2008	Shaanxi	city	NA	experienced dentists	cluster random	WHO oral health survey	412	173	41.99	_	_	_	8
**Tao.2010**[60]	2007	Anhui	city	U	trained health care doctors	stratified cluster random	Guideline for the 3rd National Oral Health Survey	1310	658	50.23	2936	156	5.31	8
**Tian et al.2009**[61]	2005	Hainan	provincial	U&R	trained experienced dentists	multiple stratified random	WHO oral health survey	720	568	78.89	3506	38	1.08	9
**Wang et al.1994**[62]	1990	Beijing	provincial	U	NA	random	NA	241	207	85.89	1334	82	6.15	8
**Wang et al.1994**[62]	1990	Sichuan	provincial	R	NA	random	NA	236	172	72.88	977	2	0.2	8
**Wang et al.1998a**[63]	1996	Shandong	city	U	trained doctors	stratified random	WHO oral health survey	3294	2095	63.6	9707	141	1.45	8
**Wang et al.1998b**[64]	1996	Shanghai	city	U	dentists	cluster	NA	848	602	70.99	_	_	_	8
**Wang et al.2001**[65]	1999	Shandong	city	U	doctors	cluster random	WHO oral health survey	1185	536	45.23	_	_	_	8
**Wang et al.2003**[66]	2000	Heilongjiang	city	U	experienced dentists	random	Guideline for the 1st National Oral Health Survey	568	349	61.44	1832	_	_	7
**Wang et al.2010**[67]	2008	Zhejiang	city	NA	trained doctors	cluster random	WHO oral health survey	928	484	52.16	2255	224	9.93	8
**Wang et al.2011a**[68]	2008	Hebei	city	U	Health care doctors	random	WHO oral health survey	6162	3650	59.23	7275	_	_	8

**Wang et al.2011b**[69]	2009	Zhejiang	city	U&R	trained dentists	cluster random	Guideline for the 3rd National Oral Health Survey	1136	773	68.05	4099	_	_	8
**Wang.1989**[70]	1988	Henan	city	U	NA	stratified cluster	criteria from <Oral Medicine>	565	437	77.35	2211	45	2.04	8
**Wei et al.2013**[71]	2012	Hubei	city	U&R	dentists	random	WHO oral health survey	435	135	31.03	526	_	_	8
**Wen et al.2013**[72]	2011	Fujian	city	U&R	trained doctors	stratified cluster random	WHO oral health survey	720	575	79.86	3774	_	_	8
**Wu et al.1995**[73]	1993	Jiangsu	city	NA	NA	stratified random	criteria from “National Survey of Student Physical Health Research Handbook”	4172	2700	64.72	10496	824	7.85	8
**Wu et al.2005**[74]	2002	Guangxi	city	NA	trained investigators	multi-stage stratified cluster random	WHO oral health survey	563	331	58.79	1545	_	_	8
**Xiao.2014**[75]	2013	Shanxi	city	U	experienced dentists	multistage stratified cluster random	Guideline for the 3rd National Oral Health Survey	217	107	49.31	543	_	_	8
**Xie et al. 2001**[76]	2000	Anhui	city	U	NA	stratified random	WHO oral health survey	567	244	43.03	927	17	1.83	8
**Xing et al.1990**[77]	1987	Zhejiang	provincial	U&R	dentists	random	NA	1088	881	80.97	_	_	_	9
**Xu et al.2010**[78]	2009	Xinjiang	city	NA	trained doctors	random	criteria from Dental Prevention Center	1101	190	17.26	_	_	_	8
**Xu et al.2013**[79]	2010	Shanghai	provincial	U	experienced dentists	multi-stage stratified random	AADA	576	106	18.4	351	0	0	7

**Cao et al.2010**[80]	2010	Shanghai	provincial	U	experienced dentists	multi-stage stratified random	AADA	600	294	49	1301	51	3.92	8
**Xu.1995**[81]	1994	Guangdong	city	NA	dentists	random	WHO oral health survey	1051	907	86.3	3434	_	_	7
**Xu.1998**[82]	1994	Fujian	city	NA	NA	random	NA	1125	968	86.04	6075	_	_	7
**Xu.2012**[83]	2010	Shanghai	provincial	U&R	dentists	multistage stratified random	WHO oral health survey	1062	603	56.78	2907	330	11.35	10
**Xu.2013**[84]	2011	Sichuan	city	U&R	trained dentists	multistage stratified random	Guideline for the 3rd National Oral Health Survey	756	415	54.89	2419	_	_	9
**Ye.2010**[85]	2008	Guangxi	city	U	dentists	random	NA	1755	1335	76.07	_	_	_	8
**Ye et al.2003(1)**[86]	1991	Hubei	city	U	dentists	cluster	WHO oral health survey	661	520	78.67	2063	_	_	8
**Ye et al.2003(2)**	1992	Hubei	city	U	dentists	cluster	WHO oral health survey	414	315	76.09	1689	_	_	8
**Ye et al.2003(3)**	1993	Hubei	city	U	dentists	cluster	WHO oral health survey	574	433	75.44	2440	_	_	8
**Ye et al.2003(4)**	1994	Hubei	city	U	dentists	cluster	WHO oral health survey	693	516	74.46	2966	_	_	8
**Ye et al.2003(5)**	1995	Hubei	city	U	dentists	cluster	WHO oral health survey	400	296	74	1648	_	_	8
**Ye et al.2003(6)**	1996	Hubei	city	U	dentists	cluster	WHO oral health survey	398	282	70.85	1576	_	_	8
**Ye et al.2003(7)**	1997	Hubei	city	U	dentists	cluster	WHO oral health survey	354	239	67.51	1260	_	_	8

**Ye et al.2003(8)**	1998	Hubei	city	U	dentists	cluster	WHO oral health survey	983	624	63.48	3242	_	_	8
**Ye et al.2003(9)**	1999	Hubei	city	U	dentists	cluster	WHO oral health survey	558	353	63.26	1713	_	_	8
**Ye et al.2003(10)**	2000	Hubei	city	U	dentists	cluster	WHO oral health survey	890	434	48.76	2630	_	_	8
**Yu et al.2006[87]**	2004	Guizhou	city	U&R	experienced dentists	Multi-stage stratified cluster	Guideline for the 2nd National Oral Health Survey	1100	608	55.27	2181	126	5.78	10
**Yu.2010[88]**	2009	Zhejiang	city	U	experienced dentists	random	Guideline for the 3rd National Oral Health Survey	1057	511	48.34	2216	_	_	8
**Zhang et al.2000[89]**	1995	Shanghai	provincial	U	trained investigators	cluster random	WHO oral health survey	12202	2897	23.74	_	_	_	8
**Zhang et al.2004[90]**	2003	Shanghai	provincial	U&R	health care doctors	random	WHO oral health survey	1965	951	48.4	4464	_	_	8
**Zhang et al.2005[91]**	2000	Liaoning	city	NA	dentists	random	WHO oral health survey	3799	1414	37.22	5941	_	_	8
**Zhang et al.2010[92]**	2009	Gansu	city	U	NA	cluster random	WHO oral health survey	4170	1427	34.22	_	_	_	8
**Zhang et al.2011[93]**	2008	Shaanxi	city	U	experienced dentists and health care doctors	random	WHO oral health survey	2626	1573	59.9	5042	408	8.09	8
**Zhao et al.2013[94]**	2012	Liaoning	city	U	experienced dentists	multi-stage stratified random	WHO oral health survey	915	589	64.37	2458	122	4.96	9
**Zhao.2000[95]**	1999	Gansu	city	U	NA	random	Guideline for the 2nd National Oral Health Survey	540	444	82.22	2718	229	8.43	8

**Zhao.2001[96]**	1998	Gansu	provincial	U&R	trained experienced dentists	random	Guideline for the 2nd National Oral Health Survey	1222	929	76.02	5302	_	_	8
**Zhong et al.2003[97]**	2001	Henan	provincial	U&R	experienced dentists	multi-stage stratified cluster	WHO oral health survey	2320	1326	57.16	5242	122	2.33	9
**Zhong et al.2011[98]**	2010	Shandong	city	U	dentists	cluster random	NA	516	195	37.79	814	4	0.49	8
**Zhou et al.2004[99]**	2002	Beijing	city	U	NA	stratified cluster random	Guideline for the 2nd National Oral Health Survey	620	369	59.52	1717	522	30.4	8
**Zhu.2003[100]**	1999	Sichuan	city	R	trained dentists	random	WHO oral health survey	4150	1118	26.94	_	_	_	8
**Zhu.2012[101]**	2011	Beijing	city	U	experienced dentists	random	Guideline for the 3rd National Oral Health Survey	7200	4874	67.69	24931	3524	14.14	8
**Zuo et al.2011[102]**	2010	Xinjiang	city	NA	dentists	cluster	Guideline for the 2nd National Oral Health Survey	1443	892	61.82	1790	_	_	8

NCOH: National Committee for Oral Health; NA: not available; U: urban; R: rural; WHO: World Health Organization; AADA: American Academy of Pediatric Dentistry; ft: number of filled teeth; dmft: number of decayed, missing and filled teeth; care index: ft/dmft%

Table S2 | Quality assessment of included 102 studies

First author & published year	Survey date	Provinces	Territorial levels	Quality score					
				total	sample population	sample size	participation rate	outcome assessment	analytical methods to control for bias
**Cai et al.2013**	2011	Shanghai	city	8	2	0	2	2	2
**Chen et al.2008**	2007	Hainan	city	8	2	0	2	2	2
**Chen.2009**	2007	Hainan	city	8	2	0	2	2	2
**Cheng et al. 2011**	2009	Anhui	city	7	2	0	2	2	1
**Chen et al.1992**	1990	Guangdong	provincial	9	2	1	2	2	2
**Cheng et al.2006**	2005	Liaoning	city	9	2	1	2	2	2
**Chen et al.2008**	2006	Zhejiang	city	8	2	0	2	2	2
**Deng et al.1998**	1995	Hunan	city	8	2	0	2	2	2
**Du et al.2007**	2001	Guangxi, Hubei	provincial	9	2	1	2	2	2
**Fan et al.2010**	2008	Guangdong	city	10	2	2	2	2	2

**Feng et al.2004**	2000	Fujian	provincial	9	2	1	2	2	2
**Feng et al.2013**	2011、2003	Shanghai	city	8	2	0	2	2	2
**Gao et al.2009a**	2007	Anhui	provincial	9	2	1	2	2	2
**Gao et al.2009b**	2008	Hebei	city	8	2	0	2	2	2
**Gao et al.2010**	2008	Xinjiang	city	8	2	0	2	2	2
**Dong et al.2013**	2012	Shandong	city	8	2	0	2	2	2
**Gou et al.2010**	2008	Chongqing	city	8	2	0	2	2	2
**Hu et al.1991**	1989	Henan	city	8	2	0	2	2	2
**Hu et al.2008**	2007	Jiangsu	city	7	2	0	2	2	1
**Hu.2013**	2012	Anhui	city	8	2	0	2	2	2
**Jiang et al.2002**	1999	Anhui	provincial	10	2	2	2	2	2
**Jiang et al.2012**	2011	Shaanxi	city	8	2	0	2	2	2
**Le et al.1992**	1992	Shanghai	city	8	2	0	2	2	2
**Li et al.1991**	1988	Hubei	city	8	2	0	2	2	2

**Li et al.1997**	1995	mainland	China	9	2	1	2	2	2
**Li et al.2007**	2006	Fujian	city	8	2	0	2	2	2
**Li et al.2010a**	2008	Fujian	city	8	2	0	2	2	2
**Li et al.2010b**	2008	Tianjin	provincial	8	2	0	2	2	2
**Li et al.2011**	2009	Shanghai	city	8	2	0	2	2	2
**Li et al.2012**	2008~2011	Shanghai	provincial	8	2	0	2	2	2
**Li et al.2013a**	2011	Hebei	city	8	2	0	2	2	2
**Li et al.2013b**	2012	Shandong	city	8	2	0	2	2	2
**Li.1998**	1996	Heilongjiang	city	7	2	0	2	2	1
**Li.2001**	1988	Jiangxi	city	8	2	0	2	2	2
**Li.2010**	2008	Gansu	city	8	2	0	2	2	2
**Li.2012**	2011	Tianjin	city	9	2	0	2	2	2
**Liang et al.2010**	2009	Guangdong	city	7	2	0	2	2	1
**Liang et al.2012**	2011	Guangdong	city	8	2	0	2	2	2
**Lin et al.2003**	2002	Chongqing	provincial	8	2	0	2	2	2
**Lin.2013**	2011	Guangdong	city	8	2	0	2	2	2
**Liu et al.1998**	1996	Guangdong	provincial	9	2	1	2	2	2

**Liu et al.2005**	1990、1996、2001	Ningxia	city	8	2	0	2	2	2
**Liu et al.2012a**	2012	Ningxia	city	8	2	0	2	2	2
**Liu et al.2012b**	2011	Sichuan	provincial	8	2	0	2	2	2
**Liu.2009**	2008	Sichuan	city	8	2	0	2	2	2
**Ma.1997**	1996	Sichuan	city	8	2	0	2	2	2
**Mao et al.1997**	1995	Zhejiang	city	9	2	1	2	2	2
**Mao et al.2011**	2010	Sichuan	city	7	2	0	2	2	1
**Meng et al.2008**	2006	Beijing	city	8	2	0	2	2	2
**NCOH.1998**	1995	China	China	10	2	2	2	2	2
**NCOH.2009**	2005	China	China	10	2	2	2	2	2
**Pei.2008**	2007	Henan	city	8	2	0	2	2	2
**Peng et al.2001a**	1997	Guangdong	city	8	2	0	2	2	2
**Peng et al.2001b**	1998	Zhejiang	city	7	2	0	2	2	1
**Peng.2001**	1998	Zhejiang	city	8	2	0	2	2	2
**Quan.1992**	1990	Hubei	city	9	2	1	2	2	2
**Sheng.2008**	2007	Liaoning	city	8	2	0	2	2	2
**Shu et al.2000**	1996	Shanghai	city	8	2	0	2	2	2

**Song et al.2011**	2008	Shaanxi	city	8	2	0	2	2	2
**Tao.2010**	2007	Anhui	city	8	2	0	2	2	2
**Tian et al.2009**	2005	Hainan	provincial	9	2	1	2	2	2
**Wang et al.1994**	1990	Beijing、Sichuan	provincial	8	2	0	2	2	2
**Wang et al.1998a**	1996	Shandong	city	8	2	0	2	2	2
**Wang et al.1998b**	1996	Shanghai	city	8	2	0	2	2	2
**Wang et al.2001**	1999	Shandong	city	8	2	0	2	2	2
**Wang et al.2003**	2000	Heilongjiang	city	7	2	0	2	2	1
**Wang et al.2010**	2008	Zhejiang	city	8	2	0	2	2	2
**Wang et al.2011a**	2008	Hebei	city	8	2	0	2	2	2
**Wang et al.2011b**	2009	Zhejiang	city	8	2	0	2	2	2
**Wang.1989**	1988	Henan	city	8	2	0	2	2	2
**Wei et al.2013**	2012	Hubei	city	8	2	0	2	2	2
**Wen et al.2013**	2011	Fujian	city	8	2	0	2	2	2

**Wu et al.1995**	1993	Jiangsu	city	8	2	0	2	2	2
**Wu.2005**	2002	Guangxi	city	8	2	0	2	2	2
**Xiao.2014**	2013	Shanxi	city	8	2	0	2	2	2
**Xie et al. 2001**	2000	Anhui	city	8	2	0	2	2	2
**Xing.1990**	1987	Zhejiang	provincial	9	2	1	2	2	2
**Xu et al.2010**	2009	Xinjiang	city	8	2	0	2	2	2
**Xu et al.2013a**	2010	Shanghai	provincial	7	2	0	2	1	2
**Xu et al.2013b**	2010	Shanghai	provincial	8	2	0	2	2	2
**Xu.1995**	1994	Guangdong	city	7	2	0	2	2	1
**Xu.1998**	1994	Fujian	city	7	2	0	2	2	1
**Xu.2012**	2010	Shanghai	provincial	10	2	2	2	2	2
**Xu.2013**	2011	Sichuan	city	9	2	1	2	2	2
**Ye.2010**	2008	Guangxi	city	8	2	0	2	2	2
**Ye et al.2003**	1991~2000	Hubei	city	8	2	0	2	2	2
**Yu et al.2006**	2004	Guizhou	city	10	2	2	2	2	2
**Yu.2010**	2009	Zhejiang	city	8	2	0	2	2	2
**Zhang et al.2000**	1995	Shanghai	provincial	8	2	0	2	2	2

**Zhang et al.2004**	2003	Shanghai	provincial	8	2	0	2	2	2
**Zhang et al.2005**	2000	Liaoning	city	8	2	0	2	2	2
**Zhang et al.2010**	2009	Gansu	city	8	2	0	2	2	2
**Zhang et al.2011**	2008	Shaanxi	city	8	2	0	2	2	2
**Zhao et al.2013**	2012	Liaoning	city	9	2	1	2	2	2
**Zhao.2000**	1999	Gansu	city	8	2	0	2	2	2
**Zhao.2001**	1998	Gansu	provincial	8	2	0	2	2	2
**Zhong et al.2003**	2001	Henan	provincial	9	2	1	2	2	2
**Zhong et al.2011**	2010	Shandong	city	8	2	0	2	2	2
**Zhou et al.2004**	2002	Beijing	city	8	2	0	2	2	2
**Zhu.2003**	1999	Sichuan	city	8	2	0	2	2	2
**Zhu.2012**	2011	Beijing	city	8	2	0	2	2	2
**Zuo et al.2011**	2010	Xinjiang	city	8	2	0	2	2	2

Table S3 | Tool for assessing the risk of bias

Bias type	Low risk (score=2)	Moderate risk (score=1)	High risk (score=0)
Selection (sample population)	● Consecutive unselected population;<br>● Sample from general population not a select group;<br>● Rationale for case and control selection explained.	● Sample selected from large population but selection criteria not defined;<br>● Sample selection ambiguous but may be representative;<br>● Eligibility criteria not explained;<br>● Rationale for cases and controls not explained;<br>● Analysis to adjust for sampling strategy bias.	● Sample selection ambiguous and sample unlikely to be representative;<br>● Highly select population making it difficult to generalise finding.
selection (sample size)	● sample size calculation conducted and adequate.	● Sample size calculation not performed but all eligible persons studied;<br>● Sample size calculation performed and reasons for not meeting sample size given.	● Sample size estimation unclear or only sub-sample studied.
Selection (participation rate)	● High participation rate (>85%).	● Moderate participation rate (70–85%).	● Low participation rate (<70%).
Performance bias (outcome assessment)	● Diagnosis using consistent criteria and direct examination.	● Assessment from hospital record or interviewer;<br>● Assessment from administrative database or register.	● Assessment from non-validated data or generic estimate from overall population.
Performance bias (analytical methods to control for bias)	● Analysis appropriate for type of sample (subgroup analysis/regression etc.).	● Analysis does not account for common adjustment.	● Data confusing.

Figure S1. Funnel plots for studies

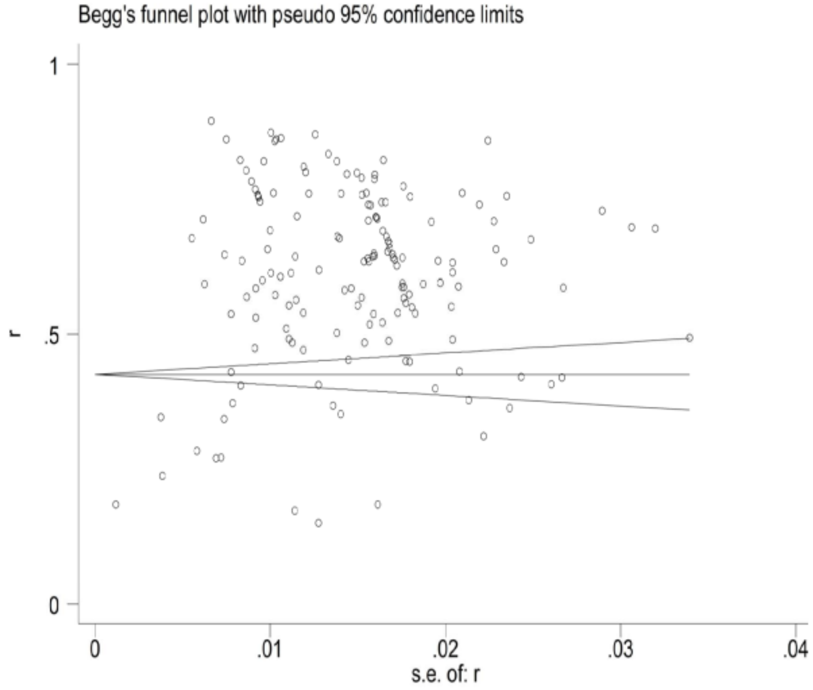

FigS1a. Funnel plot for studies of ECC prevalence

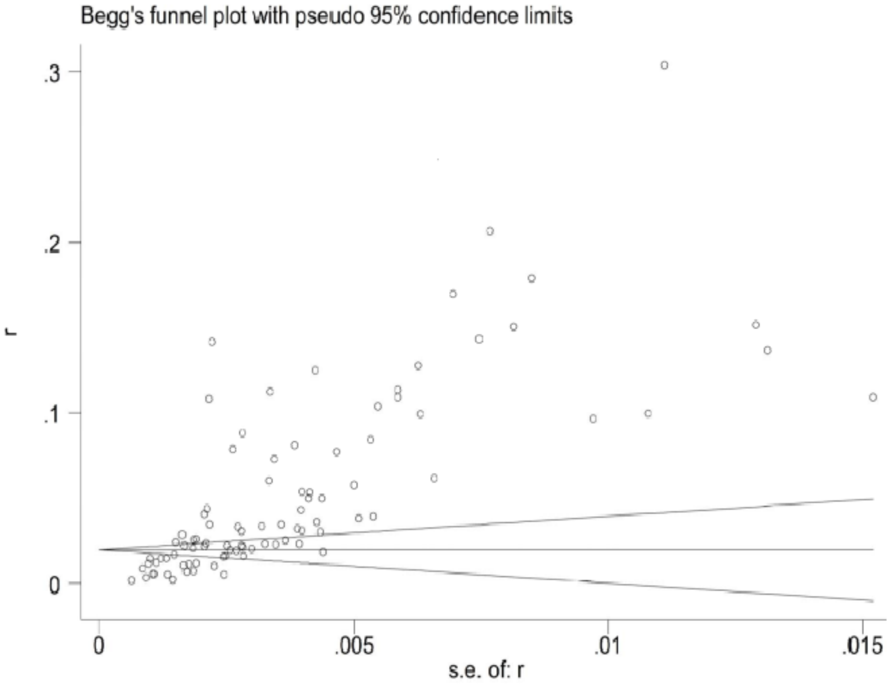

FigS1b. Funnel plot for studies of ECC care index

Checklist S1. PRISMA Checklist

Section/ topic	#	Checklist item	Reported section #
**TITLE**			
Title	1	Identify the report as a systematic review, meta-analysis, or both.	Title
**ABSTRACT**			
Structured summary	2	Provide a structured summary including, as applicable: background; objectives; data sources; study eligibility criteria, participants, and interventions; study appraisal and synthesis methods; results; limitations; conclusions and implications of key findings; systematic review registration number.	Abstract
**INTRODUCTION**			
Rationale	3	Describe the rationale for the review in the context of what is already known.	Background – 1st to 3rd paragraphs
Objectives	4	Provide an explicit statement of questions being addressed with reference to participants, interventions, comparisons, outcomes, and study design (PICOS).	Background – 4th paragraph
**METHODS**			
Protocol and registration	5	Indicate if a review protocol exists, if and where it can be accessed (e.g., Web address), and, if available, provide registration information including registration number.	N/A
Eligibility criteria	6	Specify study characteristics (e.g., PICOS, length of follow-up) and report characteristics (e.g., years considered, language, publication status) used as criteria for eligibility, giving rationale.	Methods –Selection criteria
Information sources	7	Describe all information sources (e.g., databases with dates of coverage, contact with study authors to identify additional studies) in the search and date last searched.	Methods – Search strategy
Search	8	Present full electronic search strategy for at least one database, including any limits used, such that it could be repeated.	Methods – Search strategy
Study selection	9	State the process for selecting studies (i.e., screening, eligibility, included in systematic review, and, if applicable, included in the meta-analysis).	Methods –Selection criteria
Data collection process	10	Describe method of data extraction from reports (e.g., piloted forms, independently, in duplicate) and any processes for obtaining and confirming data from	Methods – Data extraction

		investigators.	
Data items	11	List and define all variables for which data were sought (e.g., PICOS, funding sources) and any assumptions and simplifications made.	Methods –Selection criteria,Data extraction
Risk of bias in individual studies	12	Describe methods used for assessing risk of bias of individual studies (including specification of whether this was done at the study or outcome level), and how this information is to be used in any data synthesis.	Methods –Quality assessment, Table S3
Summary measures	13	State the principal summary measures (e.g., risk ratio, difference in means).	Methods – Data extraction, Statistical analysis
Synthesis of results	14	Describe the methods of handling data and combining results of studies, if done, including measures of consistency (e.g., I^2) for each meta-analysis.	Methods – Statistical analysis
Risk of bias across studies	15	Specify any assessment of risk of bias that may affect the cumulative evidence (e.g., publication bias, selective reporting within studies).	Methods – Statistical analysis
Additional analyses	16	Describe methods of additional analyses (e.g., sensitivity or subgroup analyses, meta-regression), if done, indicating which were pre-specified.	Methods – Statistical analysis
**RESULTS**			
Study selection	17	Give numbers of studies screened, assessed for eligibility, and included in the review, with reasons for exclusions at each stage, ideally with a flow diagram.	Figure 1
Study characteristics	18	For each study, present characteristics for which data were extracted (e.g., study size, PICOS, follow-up period) and provide the citations.	Results – Literature search and quality assessment, Table S1
Risk of bias within studies	19	Present data on risk of bias of each study and, if available, any outcome level assessment (see item 12).	Results –Literature search and quality scores, Table S1,S2
Results of individual studies	20	For all outcomes considered (benefits or harms), present, for each study: (a) simple summary data for each intervention group (b) effect estimates and confidence intervals, ideally with a forest plot.	Figure 2-8
Synthesis of results	21	Present results of each meta-analysis done, including confidence intervals and measures of consistency.	Table1, 2
Risk of bias across studies	22	Present results of any assessment of risk of bias across studies (see Item 15).	Results –Literature search and quality assessment

Additional analysis	23	Give results of additional analyses, if done (e.g., sensitivity or subgroup analyses, meta-regression [see Item 16]).	Results-Prevalence of ECC in mainland China, Care index of ECC in mainland China
**DISCUSSION**			
Summary of evidence	24	Summarize the main findings including the strength of evidence for each main outcome; consider their relevance to key groups (e.g., healthcare providers, users, and policy makers).	Discussion – 1st to 6th paragraphs
Limitations	25	Discuss limitations at study and outcome level (e.g., risk of bias), and at review-level (e.g., incomplete retrieval of identified research, reporting bias).	Discussion – 7th paragraph
Conclusions	26	Provide a general interpretation of the results in the context of other evidence, and implications for future research.	Discussion – 8th paragraph
**FUNDING**			
Funding	27	Describe sources of funding for the systematic review and other support (e.g., supply of data); role of funders for the systematic review.	Acknowledgements

References

1. Cai, W., Jiang, B.F. & Zhang, L.H. Survey of caries in native and non-native children located in Changning district of Shanghai. *Stomatology* **33**, 190-191 (2013).
2. Chen, C.B., Chen, H.Q. & Xing, C.Q. Caries survey report of 2354 2~6 year-old chidren in Sanya. *Maternal and Child Health Care of Chin* **23**, 4330-4332 (2008).
3. Chen, H.Q. 1866 preschool children's prevalence of dental caries in Sanya. *Maternal and Child Health Care of Chin* **20**, 2825-2826 (2009).
4. Cheng, J., Wei, S., Wang, J. & Song, D.D. The status of malnutrition among the children aged 2~5 years old and the effect factors. *Maternal and Child Health Care of Chin* **26**, 2160-2162 (2011).
5. Chen, S.X., Zhang, Y., Song, S.D., Zhang, Y.N. & Ou, Y. The comprison of people's oral health between rural and urban areas. *Acad J Guangdong Pharm Coll* **8**, 39-43 (1992).
6. Cheng, R.B., Zhang, X.F., Zhang, Y., Pan, L. & Tao, W. An epidemiological investigation of deciduous dental caries among 5375 preschool children aged between 3 to 6 years in Shenyang city. *Shanghai J Stomatol* **15**, 596-600 (2006).
7. Chen, M., Peng, S., Zheng, J. & Jiang, J.L. Survey of caries and distribution for 1160 children from 4 to 6 years old in Ningbo city. *Chin Mod Doc* **46**, 58,112 (2008).
8. Deng, Z.F., Guo, J.L. & Xu, S.S. Investigation of 2956 children's caries in Changsha. *Pract Prev Med* **5**, 241-242 (1998).
9. Du, M., Luo, Y., Zeng, X., Alkhatib, N. & Bedi, R. Caries in preschool children and its risk factors in 2 provinces in China. *Quintessence Int* **38**, 143-151 (2007).

10. Fan, W.H., Huang, S.H., Zheng, Z., Chen, W.C. & Zhang, H.C. 2008 Guangzhou children deciduous caries epidemiological survey. *J Dental Prev Treating* **18**, 127-130 (2010).
11. Feng, Y. *et al.* Fujian 5-year-old children's oral health epidemiological survey report. *J Fujian Med Univ* **38**, 87-90 (2004).
12. Feng, H. & Cai, W. 5 year-old children's caries survey report in Changning district of Shanghai. *Shanghai J Prev Med* **25**, 99-100 (2013).
13. Gao, Y.M., Han, X.L. & Yan, Y.C. The current situation of five-year-old children's caries and thier parents' oral ideas. *Acta Univ Med Anhui* **44**, 120-123 (2009).
14. Gao, S.M., Li, H.Q. & Kong, D.Q. Shijiazhuang rural plains caries survey reprot. *Hebei Med J* **31**, 1823-1824 (2009).
15. Gao, X., Xu, Y.H. & XU, P.R. Survey on health status of preschool children in Urumqi. *Maternal and Child Health Care of Chin* **21**, 3007-3009 (2010).
16. Dong, G.X., Gao, M. & Ma, C. Survey report of preschool children'scaries in Laiwu. *J of Community Med* **11**, 56-57 (2013).
17. Gou, Q.Y., Zhou, Q., Zhou, W.L., Jiang, X.Q. & Liu, L. Survey on dental caries among 3804 preschool children in Chongqing. *Maternal and Child Health Care of Chin* **25**, 2854-2855 (2010).
18. Hu, Q.Y. *et al.* Caries survey report of 1773 children in Zhengzhou. *J Henan Med Coll* **26**, 371-373 (1991).
19. Hu, Q.G. & Ren, C. Kingdergarden's children's oral health survey result in Wuxi. *Chin J of Pract Stomatol* **1**, 249 (2008).
20. Hu, D.X. Analysis on dental caries of preschool children in Tongling. *Chin J of Gen Pract* **4**, 598-599 (2013).
21. Jiang, Y., Han, X.L., Zhang, L. & Yan, Y.C. Sampling and cause analysis of five-year age groups decidudious caries prevalence and treatment needs in Anhui. *Acta Univ Med Anhui* **37**, 237-240 (2002).
22. Jiang, H. & Wang, X.Y. Invastigation and analysis of children's caries and oral health in Weinan. *Chin J Child Health Care* **20**, 474-475 (2012).
23. Le, S.X. *et al.* 1112 chilren's oral health report in Shanghai. *Stomatology* **12**, 172-174 (1992).
24. Li, Y.L. & Huang, J.M. Investigation of deciduous dental caries in preschool children in Shashi. *Hubei J Prev Med* **2**, 13-14,48 (1991).
25. Li, H., Yan, G.F. & Zhang, X. A comparative study on the incidence of dental caries of children 0~7 years in the urban and suburban rural areas of 9 cities of China. *Chin J Child Health Care* **5**, 213-215 (1997).
26. Li, R.Z., Su, R.Z. & He, Q. The investigation of caries of 1760 children aged from 2 to 7 years old in Xiamen. *Sci of travel Med* **13**, 41-42 (2007).
27. Li, Y. *et al.* Associations of social and behavioural factors with early childhood caries in Xiamen city in China. *Int J Paediatr Dent* **21**, 103-111 (2011).
28. Li, Z. & Liu, E.Q. 15490 3~6 year-old children's oral health investigation in Tianjing. *Maternal and Child Health Care of Chin* **25**, 2410-2411 (2010).
29. Li, Y.L., Du, Q. & Mao, X.Y. The epidemiological investigation into dental caries among the 5 years old in Shanghai suburban area. *Chin J of Pract Stomatol* **4**, 413-415 (2011).
30. Li, C.R., Zeng, X.L., Wang, X., Xu, W. & Chen, X. Analysis of caries status among 5-year-old children in Shanghai from 2008 to 2011. *Shanghai J Stomatol* **21**, 451-454 (2012).

31. Li, Y., Wang, L., Dong, Q. & Chen, H.W. 3~6 year-old children's caries epidemiological analysis in Tangshan. *Hebei Med J* **35**, 922 (2013).
32. Li, H., Li, A.Z., Wang, J.X. & Jiang, Y.F. The present situation of five-year-old children's caries in Linyi city and its relevant factors. *J of Shandong Med Coll* **35**, 444-447 (2013).
33. Li, M.B. 1100 preschool children's caries survey report in Jiamusi. *Heilongjiang Med and Pharm* **21**, 77-78 (1998).
34. Li, X.Q. 1234 children's caries survey report in Nanchang city. *Mod Diagn Treat* **S0**, 40 (2001).
35. Li, X.Q. Dental caries among children aged 4 to 6 investigation and analysis in Dingxi city. *Chin Pediatr of Integr Tradi West Med* **2**, 191-192 (2010).
36. Li, H. 3-5 years old children caries epidemiological analysis. *Harbin Med J* **32**, 32 (2012).
37. Liang, M.P., Li, M.Q., Li, W.Z., Chen, X.N. & Jiang, Y. Investigation and analysis of caries in Maoming kindergarten children aged 3-5. *Guide of Chin Med* **8**, 71-72 (2010).
38. Liang, C.M., Zhao, X.G., Rong, L.H., Feng, C.Z. & Li, J.F. Analysis of preschool children's caries in Jiangmen district. *Chin J Child Health Care* **20**, 761-762 (2012).
39. Lin, J.H. *et al.* Dental caries in children and adolescents of Chongqing. *J of Chongqing Med Univ* **28**, 775-777,786 (2003).
40. Lin, Q.X. Analysis of caries condition and influence factors in preschool children. *Chin Med Pharm* **3**, 75-76 (2013).
41. Liu, J.W. *et al.* Survey Result of 5~6 year-old children's caries in Guangdong Province. *J Dent Prev Treating* **6**, 12 (1998).
42. Lu, Y., Chen, D.M., Jiang, Y. & Huang, H.R. Study of dental caries prevalence in children of Yinchuan in China. *West China J Stomatol* **23**, 502-504 (2005).
43. Liu, Y., Wu, M., Zhang, X.L. & Duan, X.J. Yinchuan 5-ear-ld children deciduous teeth caries disease and related influence factors of investigation and analysis. *Harbin Med J* **32**, 421-424 (2012).
44. Liu, J., Li, X., Hu, D., Zhang, X. & He, S. Caries status of primary and permanent teeth among 6-year-old children in Sichuan province and their correlation. *West China J Stomatol* **30**, 214-216, 220 (2012).
45. Liu, J. Analysis of the related factors on deciduous dental caries in preschool children. *Chin J of Clinical Rational Drug Use* **2**, 29-31 (2009).
46. Ma, Y. Survey of 4072 Children's caries in Chengdu. *Chengdu Med* **23**, 171-172 (1997).
47. Mao, L.B. *et al.* Ningbo urban and rural residents caries epidemiology. *Mod Pract Med* **9**, 4-6 (1997).
48. Mao, K.Q. & Yang, Y.B. Sample survey on health of children aged 0~6 years old in Xichang city. *Maternal and Child Health Care of Chin* **26**, 4852-4854 (2011).
49. Meng, J. *et al.* Oral examination quality analysis of Beijing kindergarten children in rural areas. *Maternal and Child Health Care of Chin* **23**, 236-237 (2008).
50. NCOH. *Second national epideiological survey suvey of oral health* (ed Qi, J.)132-134 (People's Medical Publishing House, Beijing, 1998).
51. NCOH. *Third national epideiological survey suvey of oral health* (ed Qi, X.Q.) 60 (People's Medical Publishing House, Beijing, 2008).
52. Pei, R. Survey of preschool children's oral health in Zhengzhou. *J of Med Forum* **29**, 34-35 (2008).

53. Peng, J. *et al.* Epidemiological festures of dental caries among children and adolescent in Shenzhen. *Chin Tropical Med* **1**, 263-264 (2001).
54. Peng, C.M., Chen, H., Deng, S.L. & Peng, H. 832 3-year-old child development, nutrition and dental caries survey. *Zhejiang Prev Med* **13**, 26-27 (2001).
55. Peng, H. 3-year old children's caries investigation in Hangzhou. *Zhejiang Prev Med* **13**, 27 (2001).
56. Quan, X.H. & Liu, K.J. Investigation and analysis of caries among 598 children aged 2 to 5. *J of Mod Stomatol* **6**, 162-163 (1992).
57. Sheng, A. 4-6 years old children's oral health survey and discuss prevention strategies in Benxi City. *J of Mod Med and health* **24**, 1568-1569 (2008).
58. Shu, C.B., Feng, X.P. & Pan, Y. Investigation of deciduous teeth caries in children under 14-year-old. *Shanghai J Stomatol* **9**, 175-177 (2000).
59. Song, K.M., Yu, G.X., Zhang, F., Li, J.J. & you, S.X. Survey of dental caries incidence in 412 children aged three to five years in Xi'an city. *Chin J Conserv Dent* **21**, 101-102,109 (2011).
60. M., T.G. 5 years old Children's deciduous caries Investigation and Analysis in Tongling. *J of Tongling Vocat and Tech Coll* **1**, 4-5 (2010).
61. Tian, Y.G., Liao, T.A., Xie, Q., Zhao, X.L. & Xing, K.C. Caries prevalence among residents of Hainan province. *Chin Pub Health* **25**, 1125-1126 (2009).
62. Wang, H. *et al.* Epidemiological studies of children caries in Beijing, Leshan, HongKong and Japan. *J Beijing Med Univ* **26**, 209-212 (1994).
63. Wang, W.Y., Fu, P. & Yu, S.Y. Caries investigation of preschool children in Qingdao. *Litera and Inf On Prev Med* **4**, 193 (1998).
64. Wang, S.M., Fu, F.H., Cui, H.W., Lv, Y.M. & Zhang, J.L. Investigation of urban children's oral health. *J of Chin School Health* **19**, 94,96 (1998).
65. Wang, W.Y., Li, X.H. & He, Y.F. Caries survey report of preschool children in Qingdao. *J Improving Birth Outcome Child Develop Chin* **12**, 139-140 (2001).
66. Wang, Q., Zhao, Z.J. & Yin, F.C. 568 preschool children's oral health and caries survey report in Mudanjiang. *J of Mudanjiang Med coll* **24**, 15-16 (2003).
67. Wang, W.Q., Jing, C. & Wang, H.H. Dental caries and oral health behavior of 5-year-old children in Hangzhou city. *Stomatology* **30**, 749-750 (2010).
68. Wang, Q.C. & Wang, C.X. 2~6 year-old preschool children's caries survey report in Tangshan. *Maternal and Child Health Care of Chin* **26**, 3119-3120 (2011).
69. Wang, S.H., Zhu, S., Chen, J.P., Zhang, X. & Jiang, F. An analysis of correlative factors of deciduous teeth caries in preschool children. *Zhejiang Prev Med* **23**, 5-8 (2011).
70. Wang, X.Y. & Shi, A.M. Caries survey among children in Zhengzhou aged 3 to 6. *J Appl Clin Pediatr* **4**, 368-371 (1989).
71. Wei, Y.H., Wang, F. & Xu, W. Investigation of preschool children's caries. *J Clin Stomatol* **29**, 681-682 (2013).
72. Wen, Y.J. *et al.* Analysis of five-year-olds caries status and related factors in Ningde city of Fujian Province. *Chin J Prim Med Pharm* **20**, 3589-3591 (2013).
73. Wu, S.Y., Yu, J. & Xu, D.N. Epidemiological survey of 10491 children's oral health in Changzhou city. *Stomatology* **15**, 100-101 (1995).
74. Wu, W., Zeng, X.J. & Huang, H. Survey reasult of 3~5 year-old children's caries in Nanning, Guangxi province. *J of Mod Stomatol* **19**, 407 (2005).

75. Xiao, Y. Investigation of preschool children's caries. *For all Health* **8**, 295 (2014).
76. Xie, X.M. 567 preschool children aged 3 to 7 deciduous caries survey. *Acta Univ Med Anhui* **36**, 321 (2001).
77. Xing, X.L., Wu, J.C., Jin, G.R., Zheng, K.H. & Zhu, Q.Z. Investigation of 12286 children's caries. *J of Zhejiang Med Univ* **19**, 280-282 (1990).
78. Xu, Y.H., Li, Y.P. & Chen, C.H. Karamay city preschool children's health collective survey. *World Health Digest Med Periodical* **7**, 376-377 (2010).
79. Xu, S.T., Cao, X.M., Feng, J.Q., Li, C.R. & Song, Y. Analysis of early childhood caries and risk factors in 2 year-old children in Shanghai. *J Dental Prev Treating* **21**, 301-304 (2013).
80. Cao, X.M., Xu, S.T., Feng, J.Q., Dong, H.W. & Li, C.R. Status of early childhood caries of 3 year-old children in Shanghai. *J Dent Prev Treating* **18**, 643-646 (2010).
81. Xu, Y.C. 1051 preschool children's caries survey result in Jieyang city. *J Dent Prev Treating* **3**, 54 (1995).
82. Xu, M.Y. Investigation of the incidence of dental caries in preschool children. *Chin J Child Health Care* **6**, 217 (1998).
83. Xu, W. Risk Factors for Dental Caries in 3 -year-old and 6-year-old Children in Shanghai with the Preventive Strategies Research. *Fudan Univ J* **5**, 20-25(2012).
84. Xu, B.Q., Wen, X.Y. & Guo, H.J. Investigation of dental caries of 5-year-old children and their parents' oral hygienic knowledge and behavior in Mianyang. *Maternal and Child Health Care of Chin* **23**, 3815-3817 (2013).
85. Ye, Y. Survey reault of 3~6 year-old children's caries in Guigang, Guangxi province. *National Med Front of Chin* **5**, 96 (2010).
86. Ye, Y., Li, P. & Li, X.T. Shiyan City caries in preschool children 10 years of observation. *Chin J Child Health Care* **11**, 141-142 (2003).
87. Yu, X.Y. & Ma, L.X. Guiyang 5-year-old child deciduous caries sample survey report. *Guizhou Med J* **30**, 659-660 (2006).
88. Yu, J. 3~6 year-old children's caries survey result in Hangzhou. *Zhejiang Prev Med* **22**, 67,70 (2010).
89. Zhang, J., Wei, M. & Zhang, Y. Caries investigation of children in Shanghai below 7 and its trend in 10 years. *Shanghai J Prev Med* **12**, 385-386 (2000).
90. Zhang, J.M., Zhu, J.F. & Lu, Y.P. Investigation and treatment of dental caries in preschool children. *Anthology of Med* **23**, 428-430 (2004).
91. Zhang, X.F., Xu, X.B., Cheng, R.B. & Pan, L. Study of caries polarization in 2-5 year-old children of Shenyang, China. *West China J Stomatol* **23**, 258-259 (2005).
92. Zhang, C.Y. & Zhang, Y.F. Preschoolers physical development trends, characteristics and status dynamic analysis. *Health Vocat Edu* **28**, 100-101 (2010).
93. Zhang, P., Yang, T.F. & Li, Y.Q. 2626 chidren aged 2 to 6 's caries survey in Weinan. *Shaanxi Med J* **40**, 1372-1373 (2011).
94. Zhao, P.Y. *et al.* Status of caries of 3 year-old children in shenyang. *Chin Med Eng* **21**, 3-5 (2013).
95. Zhao, W.H. 5-year-old child deciduous caries sample survey report in Lanzhou. *Gansu Sci and Tech* **4**, 48 (2000).
96. Zhao, W.H. Caries investigation and analysis of urban and rural, poor areas in Gansu Province. *Chin J Conserv Dent* **11**, 121-122 (2001).

97. Zhong, Y., Yang, B.S. & He, J. Investigation of deciduous caries in preschool children in Henan. *Henan J Prev Med* **14**, 141-143 (2003).
98. Zhong, L.H. *et al.* Caries epidemiological invastigation and analysis of children aged 3 to 4. *Guide of Chin Med* **9**, 79-80 (2011).
99. Zhou, H.L. *et al.* Statistics and analysis of Xicheng oral health survey. *Beijing J Stomatol* **12**, 160-162 (2004).
100. Zhu, Q.H. Caries investigation and analysis of rural children in Bazhong. *Chin Gen Pract* **6**, 137-138 (2003).
101. Zhu, Z.G. Caries survey analysis of preschool children in Changping area, Beijing City, 2011. *J of TCM Univ of Hunan* **32**, 67-68 (2012).
102. Zuo, Z.Y. & Chen, H. Investigation and analysis of preschool children's caries. *Chin and foreign Med Res* **9**, 73 (2011).
